# Keeping the Distance: Activity Control in Solid-Supported Sucrose Phosphorylase by a Rigid α-Helical Linker of Tunable Spacer Length

**DOI:** 10.1021/acscatal.4c05616

**Published:** 2024-11-06

**Authors:** Chao Zhong, Anisha Vyas, Jakob D. H. Liu, Chris Oostenbrink, Bernd Nidetzky

**Affiliations:** 1 Institute of Biotechnology and Biochemical Engineering, Graz University of Technology, NAWI Graz, Petersgasse 12, Graz 8010, Austria; 2 Austrian Centre of Industrial Biotechnology (ACIB), Krenngasse 37, Graz 8010, Austria; 3 Institute of Molecular Modeling and Simulation, University of Natural Resources and Life Sciences (BOKU), Muthgasse 18, Vienna 1190, Austria

**Keywords:** α-helical peptide linker, molecular spacer, enzyme immobilization, sucrose phosphorylase, molecular modeling, thermodynamic analysis, enthalpy−entropy compensation

## Abstract

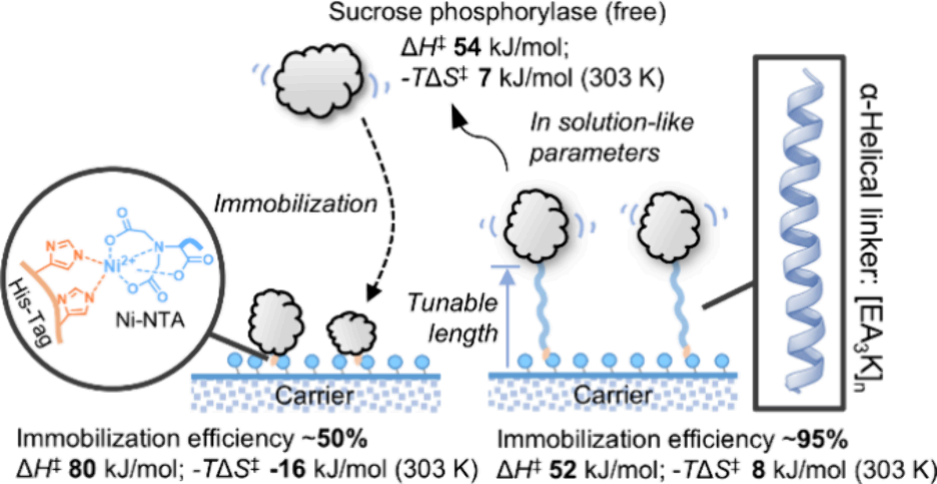

Enzyme immobilization into carrier materials has broad importance in biotechnology, yet understanding the catalysis of enzymes bound to solid surfaces remains challenging. Here, we explore surface effects on the catalysis of sucrose phosphorylase through a fusion protein approach. We immobilize the enzyme via a structurally rigid α-helical linker [EA_3_K]_*n*_ of tunable spacer length due to the variable number of pentapeptide repeats used (*n* = 6, 14, 19). Molecular modeling and simulation approaches delineate the conformational space sampled by each linker relative to its His-tag cap used for surface tethering. The population distribution of linker conformers gets broader, with a consequent shift of the enzyme-to-surface distance to larger values (≤15 nm), as the spacer length increases. Based on temperature kinetic studies, we obtain an energetic description of catalysis by the enzyme-to-linker fusions in solution and immobilize on Ni^2+^-chelate agarose. The solid-supported enzymes involve distinct changes in enthalpy–entropy partitioning within the frame of invariant Gibbs free energy of activation (Δ*G*^‡^ = ∼61 kJ/mol at 30 °C). The entropic contribution (−*T*Δ*S*^‡^) to Δ*G*^‡^ increases with the spacer length, from −16.4 kJ/mol in the linker-free enzyme to +7.9 kJ/mol in the [EA_3_K]_19_ linked fusion. The immobilized [EA_3_K]_19_ fusion protein is indistinguishable in its catalytic properties from the enzymes in solution, which behave identically regardless of their linker. Enzymes positioned closer to the surface arguably experience a higher degree of molecular organization (“rigidification”) that must relax for catalysis through the additional uptake of heat, compensated by a gain in entropy. Increased thermostability of these enzymes (up to 2.8-fold) is consistent with the proposed rigidification effect. Collectively, our study reveals surface effects on the activation parameters of sucrose phosphorylase catalysis and shows their consistent dependence on the length of the surface-tethering linker. The fundamental insight here obtained, together with the successful extension of the principle to a different enzyme (nigerose phosphorylase), suggests that rigid linker-based control of the protein–surface distance can be used as an engineering strategy to optimize the activity characteristics of immobilized enzymes.

## Introduction

Enzymes are exceptionally efficient catalysts of chemical reactions.^1−3^ Technologies surrounding their use have a huge importance in modern industrial production^4−8^ as well as broadly across the medical sector.^5^ Immobilization (i.e., the incorporation of enzymes into solid material to generate a catalytically functional composite) is central to the development of a large number of enzyme applications.^9−12^ Despite its immense relevance as a practical principle, immobilization still involves a remarkable lack of understanding of the governing mechanisms, especially at the molecular level.^9,13−15^ In particular, how enzyme interactions at the interface with solid materials affect catalysis is a fundamental question that remains as yet unexplained.^13,15−17^ Experimental study of enzyme catalysis under the confinement of solid surfaces confronts the major challenge that the observable behavior of an immobilized enzyme is affected by multiple factors, including conformational rearrangements of variable type and degree, that often overlap in effect and are extremely difficult to disentangle.^9,13−15,18^ Interaction with the solid surface can furthermore introduce substantial heterogeneity into the enzyme population.^14,15^ Heterogeneity arises from nonuniform modes of enzyme binding to the surface and results in differences in the rotational and configurational space accessible to the individual enzyme molecules in their respective immobilized states.^18−20^ While advanced biophysical methods have greatly extended the limits of monitoring basic features of protein structure and activity at solid surfaces,^13,21,22^ some even at single-molecule resolution,^23−26^ none is immediately pertinent to the characterization of surface effects specifically on the catalysis of an immobilized enzyme. To pursue our inquiry, we realized the need for progress in immobilization methodology to achieve control over the enzyme-surface interactions in two crucial respects: first, the site specificity of surface tethering of the enzyme and second, adjustment of the protein-to-surface distance. Distance control is particularly challenging to implement in immobilized enzymes, and we here present a fusion protein approach to its accomplishment.^27^ Considering the evidence that solids can extend into the liquid bulk by ∼10 Å via layers of ordered water adjacent to the surface,^28,29^ it becomes clear that a controlled modulation of enzyme-to-surface distance is critical for the study of surface effects on catalysis. Site-specific tethering on the other hand is critical to minimize undesired enzyme–surface interactions and to restrict population heterogeneity in the immobilized preparation of enzymes.^24^

Our choice of strategy to adjust the enzyme-to-surface distance took into consideration the options presented by chemical coating of the solid surface.^15,18^ There exist numerous methods to tune protein–surface interactions through the use of surface-assembled monolayers, polymer layers, or other types of surface modification. However, despite their high importance in making solid surfaces better compatible with the requirements of the enzyme used, surface layers merely extend the solid surface. They do not constitute true “spacers” in the sense of modulating the distance between the solid surface and the enzyme tethered to it.^30−32^ A genetically encoded peptide linker suitably fused to the enzyme of interest appeared to be a more promising candidate to fulfill the role of a molecular spacer in immobilization.^27^

Peptide linkers are commonly found in natural proteins of modular structural organization. Their role is to join the component modules and enable dynamic interactions between them.^33^ Selection of a suitable peptide linker is thus critical in fusion protein-based approaches of protein engineering.^34,35^ Peptide linkers are broadly categorized as being structurally rigid or flexible. Whereas cooperative interaction among protein modules often necessitates a peptide linker of sufficient flexibility, the role of the structural spacer in enzyme immobilization can only be fulfilled by a rigid linker.^33−35^ Peptide linkers can acquire rigidity by folding into an α-helical structure.^36,37^ Proline-rich linkers evidently cannot form an α-helix but are somewhat rigid from their sequence intrinsically.^38^ Considering the task of adjusting the enzyme-to-surface distance, programmability and tunability of the spacer length are critical features. We reasoned that only structurally well-organized (i.e., folded) linkers could provide them in an appropriate manner.^33,35,38^

Here, we report the realization of the above ideas based on a linker fusion approach applied to the enzyme sucrose phosphorylase (EC 2.4.1.7). The main reason for selecting sucrose phosphorylase was the judicious choice of a model enzyme (Scheme S1). The sucrose phosphorylase is well-characterized biochemically and mechanistically.^39−42^ The enzyme is generally stable, it can be produced well in *Escherichia coli* and is known for facile immobilization.^43−46^ There is broad interest in sucrose phosphorylase within the field of applied biocatalysis.^43,47^ The enzyme is reported for the synthesis of diverse glucosides and saccharides.^43,48−50^ Industrial processes for the production of glucosylglycerol^51−53^ and cellobiose^54−56^ employ sucrose phosphorylase in a soluble or carrier-bound immobilized form. To construct fusion proteins of sucrose phosphorylase, we considered the pentapeptide [EA_3_K], which is a known α-helical type of rigid linker.^37^ Its fusion in multiple repeats (*n* = 6–19) to the enzyme provided a tunable spacer length (up to ∼15 nm^57^) for immobilization of the sucrose phosphorylase at variable distances from the carrier surface. A mixed molecular dynamics (MD) and molecular modeling approach outlines the accessible conformational space sampled by each linker in the immobilized fusion protein. The population distribution of modeled linker conformers gets broader and shifts to larger median values of the enzyme-to-surface distance as the nominal spacer length increases. Biochemical studies show that the immobilized sucrose phosphorylase gradually acquires characteristics (activity and stability) of the linker-free enzyme in solution as the distance to the surface increases. Temperature kinetic studies reveal subtle effects of the surface on enzyme catalysis. The Gibbs free energy of activation is unchanged in all enzymes, regardless of their linker and whether they are soluble or immobilized on the carrier. The immobilized enzymes, however, exhibit a change in enthalpy–entropy partitioning compared with the enzymes in solution. The effect on the thermodynamic activation parameters becomes stronger the closer the sucrose phosphorylase is positioned to the surface. The results are explained in terms of an enhanced degree of molecular organization (“rigidification”)^13,20,58^ experienced by the enzymes due to the interaction with the solid surface and the water layers attached to it. The rigidified enzyme conformation must relax for catalysis through the additional uptake of heat, compensated by a gain in entropy. The proposed rigidification is further supported by the difference in thermostability of the immobilized enzymes dependent on their linker length. The fundamental insight obtained here identifies the rigid linker-based control of the protein–surface distance as a promising engineering strategy to optimize the activity characteristics of immobilized enzymes. The notion is further confirmed by applying the linker approach to a second enzyme, namely nigerose phosphorylase (EC 2.4.1.279).^59^

## Experimental Section

### Materials

Unless otherwise stated, all chemicals were from Sigma-Aldrich (Vienna, Austria) or Carl Roth (Karlsruhe, Germany). Codon-optimized genes of peptide linkers (for sequence details, see the Supporting Information and Section S1.1) were from GenScript Biotech (Leiden, Netherlands). Ni-NTA Superflow nickel-charged resins (agarose beads) in a size of 60–160 μm were from QIAGEN GmbH (Düsseldorf, Germany). The beads were stored in aqueous ethanol (20 vol %) at a concentration of 20 mg dry mass/mL.

### Enzymes

Sucrose phosphorylase from *Leuconostoc mesenteroides* (*Lm*SP; GenBank no. BAA14344.1) and nigerose phosphorylase from *Anaerosporobacter mobilis* (*Am*NP; GenBank no. SHM33122.1) were used. Both enzymes were obtained with N- or C-terminal His-tag,^52^ or with N-terminal Strep-tag II (for *Lm*SP^60^). Unless otherwise stated, the C-terminally His-tagged *Lm*SP and *Am*NP were used as enzyme references throughout.

For both *Lm*SP and *Am*NP, sequences encoding the peptide Glu-Ala-Ala-Ala-Lys in multiple repeats (*n*) were positioned either at the 5′- or 3′-end, immediately downstream (*Lm*SP: *n* = 7; *Am*NP: *n* = 6, 12) or upstream (*Lm*SP: *n* = 6, 14, 19; *Am*NP: *n* = 14, 17) of the His-tag sequence. To join the His-tag and the linker, the peptide Leu-Ala was included in the N-terminal linker-fused constructs and Ala-Ala-Ala-Leu-Glu was included in the C-terminal linker-fused constructs. The cloning work is described in the Supporting Information (Section S1.2; for primers used, see Tables S1 and S2). The coding gene of *Am*NP was kindly supplied by Prof. Tom Desmet (Ghent, Belgium). The vector pET-21b(+) was used for the expression of the linker constructs of *Lm*SP and *Am*NP. The N-terminally His-tagged and Strep-tagged *Lm*SP was expressed using the pQE30 and pASK-IBA7+ plasmid, respectively.

Enzymes were produced and purified by standard procedures (Supporting Information, Section S1.3), and their purity was verified by SDS-PAGE. Purified *Lm*SP constructs were stored in sodium phosphate buffer (50 mM, pH 7.4) containing 50 mM NaCl. Purified *Am*NP constructs were stored in MES buffer (50 mM, pH 7.2) containing 50 mM NaCl. Protein concentration was determined with the RotiQuant reagent (Carl Roth) employing bovine serum albumin (BSA) as the standard. Alternatively, it was determined by measurement of absorbance at 280 nm using a NanoPhotometer N50 (Implen, Munich, Germany). The molar extinction coefficient and molecular mass were calculated with the ExPASy ProtParam tool:^61^*Lm*SP (75750 M^–1^ cm^–1^, 56807.6 Da) and *Am*NP (138550 M^–1^ cm^–1^, 85165.3 Da). Both methods for protein concentration determination provided consistent results (Table S3).

### Protein Modeling Supported by Molecular Dynamics Simulation

**Figure 1:**
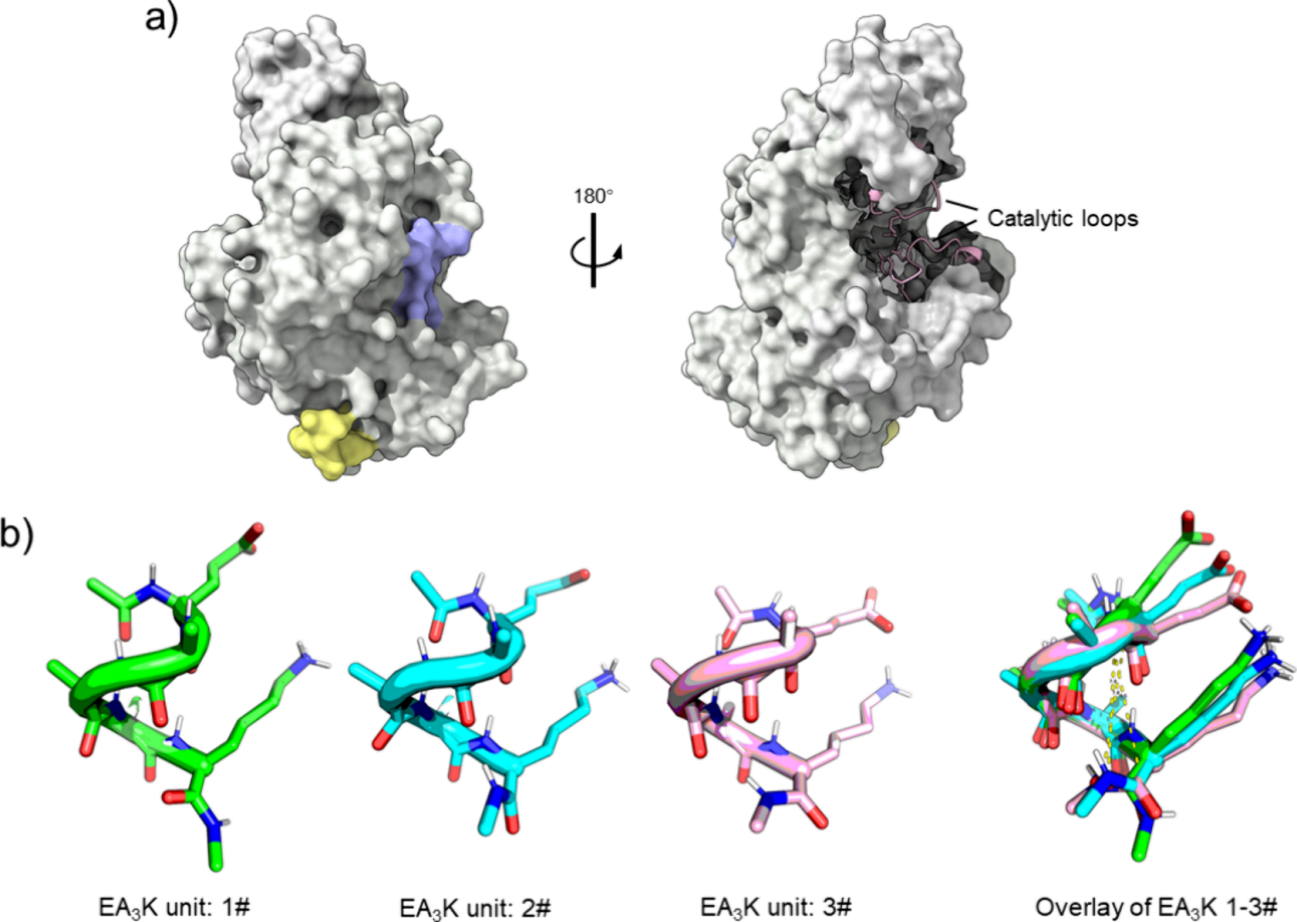
Structure models of *Lm*SP and the linker attached to the enzyme. (a) AlphaFold structure model of *Lm*SP. Side views are related by an 180° rotation around the vertical axis (shown by Chimera X). The N- and C-termini of enzyme are colored in purple and yellow, respectively. (b) Representative structures of the three main clusters of the EA_3_K unit used to construct the [EA_3_K]_*n*_ linkers, with the overlay of their structures shown in the right panel. The N-to-N′ distance, i.e., distance from the first N (residue) in the backbone down to the last N′ where the N of the next unit is fused, is estimated to be 0.95, 0.78, and 0.79 nm for cluster 1–3#, respectively.

The *Lm*SP structure was modeled using AlphaFold Monomer v2.0 (serial number AF-Q59495-F1; Figure 1a).^62^ Linker modeling followed a hierarchical approach using GROMOS11 simulation software. Atomic interactions were calculated based on the GROMOS 54A8_bb force field.^63^ The EA_3_K fragment was solvated in a simple point-of-charge water environment with 0.1 M NaCl. The simulation box size was set to maintain a 1.4 nm minimum distance between the peptide and box walls. Ten replicate simulations were performed, each with different initial velocities and a duration of 100 ns. Afterward, all trajectories were jointly clustered, revealing three primary clusters that accounted for the majority of the conformational states (Figure 1b and details in the Supporting Information, Section S1.4). The central member structures of these clusters were then used to model the [EA_3_K]_*n*_ linkers.

Modeling [EA_3_K]_*n*_ fragments involved constructing polymers incrementally in sets of 6 units (*n* = 6, 12, 18) to streamline the process instead of the experimentally used *n* = 6, 14, and 19. The assembly initiated with a fusion of three distinct EA_3_K clusters, forming nine distinct [EA_3_K]_2_ units. Sequential elongation by appending three [EA_3_K]_1_ units eventually generated 729 unique six-unit combinations. For 12-unit structures, the modeled 6-unit fragments were grouped into 25 clusters that were paired to form 625 of 12-unit polymers, which were once again clustered into 25 clusters. Another 25 of the six-unit fragments were added to form the 18-unit fragments in 625 models.

Each fusion step involved energy minimization to resolve minor steric clashes, followed by excluding configurations with nonbonded interaction energies that are above a zero-threshold value. All three linker variations had the A_3_LE peptide appended to the C-terminus to link to His-tag, with the N-terminus attached to the C-terminus of *Lm*SP. Aligning structures on the A_3_LE backbone facilitated simultaneous modeling of all potential states and calculation of protein-to-surface distance, assuming that the surface was at the level of the C-terminus of the A_3_LE fragment, perpendicular to the helical axis. The vertical distance from the enzyme’s center of geometry to the surface, minus the radius of gyration, serves as an approximation for the distance from the surface to the nearest point on the enzyme’s surface.

### Enzyme Immobilization

For the immobilization of the *Lm*SP constructs, Ni-NTA beads were washed with 50 mM potassium phosphate buffer (pH 7.0) before use. Immobilization was performed in a total liquid volume of 1.0 mL. The enzyme was diluted to ∼2.0 mg/mL and mixed with varying amounts of the carrier (up to 10 mg dry mass). The suspension was incubated in an end-over-end rotator (Stuart SB3, Cole-Parmer, Stone, UK) at 40 rpm and 25 °C for 1.5 h. A sample was taken from the supernatant after centrifugation (9400*g*, 4 °C, 5 min) for the protein concentration assay. The recovered beads were washed twice with phosphate buffer (50 mM, pH 7.0) and stored at 4 °C until use.

To perform *Lm*SP immobilization on a plain carrier lacking chelated Ni^2+^ groups, the beads were suspended in 20 mM phosphate buffer (pH 7.4) containing 50 mM EDTA and 0.5 M sodium chloride. They were incubated for 30 min on the end-over-end rotator at 40 rpm and 25 °C. The washed beads (with 50 mM phosphate buffer, pH 7.0) were then used as described above.

Immobilization of the *Am*NP constructs was done analogously as for the *Lm*SP constructs, except for the following modifications: Ni-NTA beads were washed with 50 mM MES buffer (pH 7.2) before use. The enzymes were diluted to approximately 0.5 mg/mL and mixed with 10 mg (dry mass) of the carrier. The suspension was incubated in an end-over-end rotator at 25 rpm and 25 °C for 1.5 h. The beads were then washed twice with MES buffer (50 mM, pH 7.2) and stored at 4 °C until use.

The immobilization yield (*Y*, %) was determined with the relationship *Y* = 100 × (*c*_0_ – *c*)/*c*_0_, where *c*_0_ and *c* are the concentration of enzyme in solution before and after immobilization, respectively. The immobilization efficiency (η, %) was determined with the relationship η = 100 × *a*_m_/*a*_c_, where *a*_m_ and *a*_c_ are the measured and calculated activities on the carrier (U/g), respectively. *a*_c_ is calculated from the bound protein concentration (*c*_0_ – *c*) and the known specific activity of the enzyme (U/mg).

### Activity Assay

The activity assay used was the same for the free and immobilized enzymes. For the *Lm*SP constructs, the conversion of sucrose and phosphate was analyzed by measuring the released α-glucose 1-phosphate. Incubations were done in 1.0 mL of liquid at 30 °C, using a ThermoMixer C (Eppendorf, Vienna, Austria) for temperature control and agitation at 1000 rpm. The substrate solution contained 50 mM of each sucrose and phosphate buffer (pH 7.0). Both substrate concentrations were saturated for the enzyme at a steady state. BSA (0.1 mg/mL) was added to stabilize *Lm*SP in the conditions of the assay performed at low enzyme concentrations.^41^ Compared to earlier results of the C-terminally His-tagged *Lm*SP,^52^ the stabilization resulted in a 1.6-fold enhanced specific activity (see later under Results and Discussion). Homogeneous samples (200 μL) were taken at appropriate intervals (≥3 times) within 6 min, heated at 99 °C for 5 min, and centrifuged (9400*g*, 4 °C, 5 min). The α-glucose-1-phosphate released was determined with a coupled enzymatic assay,^41^ with absorbance from NADH measured on a FLUOstar Omega plate reader (BMG Labtech, Ortenberg, Germany). The activity unit (U) refers to 1 μmol of NADH/min under the condition used.

The activity of the *Am*NP constructs was measured in the direction of nigerose synthesis from 20 mM β-glucose 1-phosphate (provided by Prof. David Wilson, University of California, San Francisco, USA) and 50 mM glucose in MES buffer (50 mM, pH 7.2). Both substrates were saturating for the enzyme at a steady state.^59^ Incubations were done in 0.2 mL volume at 37 °C. Homogeneous samples (40 μL) were taken at intervals (≥3 times) over 7 min, heated at 99 °C for 5 min, and centrifuged (9400*g*, 4 °C, 5 min). The phosphate released was measured using the colorimetric assay by Saheki et al.^64^ The activity unit (U) is defined as the amount of enzyme producing 1 μmol of phosphate/min under the conditions used.

### Temperature Kinetics

Reactions were performed with soluble and immobilized enzymes exactly as described above, except variable temperatures were applied (*Lm*SP: 10–35 °C; *Am*NP: 20–33 °C). For temperatures below 20 °C, the ThermoMixer C (with temperature set) was placed in a cooling room (∼4 °C). Reactions were run for either 6 min (≥20 °C) or 30 min (≤15 °C). Homogeneous samples were taken periodically (≥3 times) and the initial rate (*V*) was determined from the linear time courses of product release (*Lm*SP: α-glucose-1-phosphate; *Am*NP: phosphate) in triplicate. The catalytic constant *k*_cat_ was calculated with the relationship *k*_cat_ = *V*/*E*, where *E* is the molar enzyme concentration. *E* is determined from the protein concentration and molar mass of the enzyme used. *V* is assumed as the maximum rate at all temperatures, considering that the substrate concentration was saturating at the highest temperature used. The temperature dependence of *k*_cat_ was described with the Eyring model (eq 1):
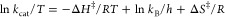
1

Δ*H*^‡^ is enthalpy (J/mol), Δ*S*^‡^ is entropy (J/(mol·K)), *T* is absolute temperature (K), *R* is the ideal gas constant (8.314 J/(mol·K)), *k*_B_ is Boltzmann’s constant (1.3806 × 10^–23^ J/K), and *h* is Planck’s constant (6.6261 × 10^–34^ J·s). Linear least-squares fitting was done with Origin 2019b. The reported fits show correlation coefficients of 0.98 or greater and involve reasonably normal distributions of the residuals.

### Stability

This assay was applied to the *Lm*SP constructs. Enzyme stability was determined from the irreversible loss of activity at 40 °C.^44^ The enzyme (∼10 μg/mL) was incubated in phosphate buffer (50 mM, pH 7.0) with agitation at 1000 rpm. The residual activity was assayed in samples (≥5) taken at certain times over a representative time to give ≥50% loss of initial activity. The deactivation constant *k*_D_ (h^–1^) was obtained from linear semilog plots of residual activity versus incubation time.

## Results and Discussion

### Peptide Linker Fusions of Phosphorylases

**Figure 2:**
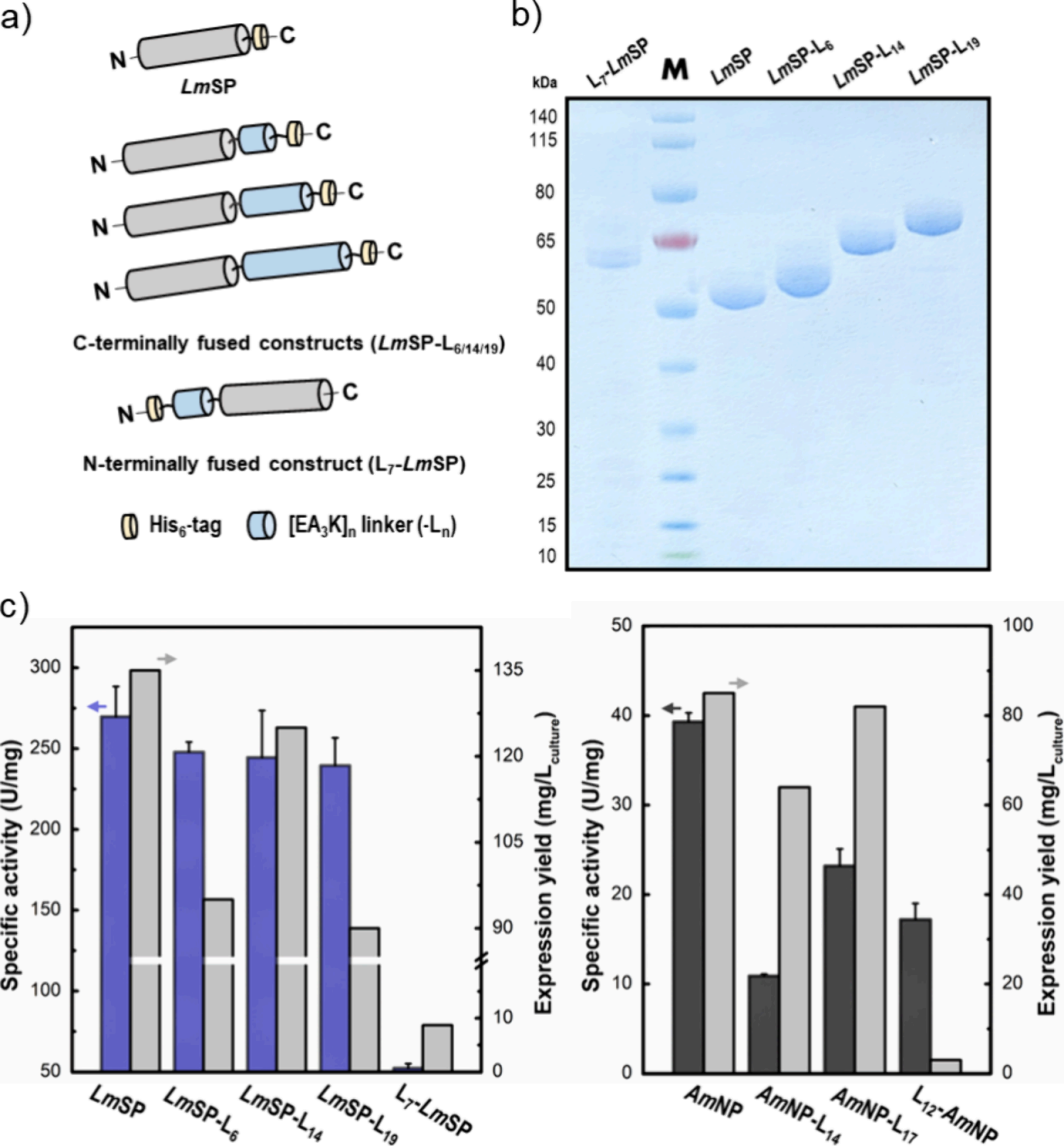
Linker fusions used. (a) *Lm*SP constructs with the His-tagged [EA_3_K]_*n*_ linker fused to either N- or C-terminus. (b) Purified preparations of native *Lm*SP and N-/C-terminal linker fusion constructs thereof. M, PageRuler Prestained Protein Ladder (10–140 kDa); the purified linker-fusion proteins migrated to positions in the gel consistent with their expected molecular masses, except for L_7_-*Lm*SP (60.4 kDa) that showed a slightly higher apparent molecular mass. (c) Expression yield (mg/L_culture_) and specific activity (U/mg; *N* ≥ 2) of *Lm*SP constructs (left panel) and *Am*NP constructs (right panel).

Sucrose phosphorylases occur naturally as functional monomers or homodimers.^43^ To avoid complications due to quaternary protein organization, we selected the monomeric *Lm*SP (Figure 1a). Among the possible choices of a rigid linker (Figure S2), we opted here for the pentapeptide EA_3_K used in a variable number (*n*) of repeats. The [EA_3_K]_*n*_ type of linkers is known to adopt α-helical conformation under strict structural restraint.^37^ They have been used previously with different enzymes to construct (multi)functional fusion proteins (Table S4).^33,37^ The linker length can be adjusted conveniently by the *n* repeats with overall retention of the α-helix structure.^57^ The surface of [EA_3_K]_*n*_ α-helix linkers is relatively neutral in terms of polarity and charge,^37^ which arguably minimizes the risk of undesired linker interactions with the fusion partner enzyme, the carrier surface, or both. Overall, these linker properties were promising in facilitating an outward extension of the immobilized enzyme from the carrier surface. Linkers were fused to the N- or C-terminus of *Lm*SP. Both termini are readily accessible in the protein structure (Figure 1a) and have previously been appended with the His-tag without negative effect on the activity.^52^ All *Lm*SP linker constructs were equipped with a His-tag cap that served as the surface tethering group for site-selective enzyme immobilization on solid carriers. Note that the use of the [EA_3_K]_*n*_ linkers is not limited to the His-tag: the maltose binding protein^65^ and the SpyTag^66^ have also been applied with other proteins (Table S5), suggesting general flexibility in the design of linker-tag combinations for immobilization. In the constructs used here, the His-tag was connected to the main linker by an unstructured pentapeptide (A_3_LE; Figure 2a). The triple Ala insertion between linker and tag was chosen based on earlier works describing [EA_3_K]_*n*_ linkers.^67,68^ The Leu-Glu extension is derived from the *Xho*I restriction site of the pET21b(+) vector used. We refer to the fusion proteins as L_n_-*Lm*SP and *Lm*SP-L_n_ where L (linker) before or after *Lm*SP indicates the N- or C-terminal position, respectively, and index n shows the *n* repeats of EA_3_K.

Additionally, the linker approach was extended to *Am*NP.^59^ Like *Lm*SP, *Am*NP is a functional monomer.^59^ Both N- and C-termini are exposed on the protein surface (Figure S3) and appear to be suitable for the attachment of a structured linker. The fusion proteins L_n_-*Am*NP and *Am*NP-L_n_ were constructed by using the approach described above for *Lm*SP.

### Characterization of the Linker Fusions

We first examined L_7_-*Lm*SP and *Lm*SP-L_6_ (Figure 2). Whereas *Lm*SP-L_6_ was expressed to a similar level in *E. coli* (∼100 mg/L culture) as the linker-free reference enzyme (∼130 mg/L culture), L_7_-*Lm*SP exhibited lower expression levels (<10 mg/L culture). As both N- and C-termini of *Lm*SP appear to meet the basic structural requirements to accommodate the linker, the large difference in the expression of L_7_-*Lm*SP and *Lm*SP-L_6_ was unexpected. *Lm*SP constructs with only the His-tag at the N- and C-termini were expressed at similar levels. A similar trend was observed with *Am*NP: the N-terminal linker construct (here: L_12_-*Am*NP; < 5 mg/L) was expressed much less efficiently than the relevantly similar C-terminal linker constructs (here: *Am*NP-L_14_, *Am*NP-L_17_; ≥ 60 mg/L). A plausible explanation for the observed differences in enzyme expression is that the α-helical linker interferes with protein folding, in particular, when it is positioned at the N-terminus. However, we also considered that the N-terminal linkers with multiple pentapeptide repeats might overburden the translational machinery of the cell, thereby reducing the level of protein production. Indeed, there is evidence from the studies on other peptide repeat linkers^69,70^ that the supply of loaded tRNA can become limiting for the production of N-terminal linker constructs whereas the effect is less pronounced in the corresponding C-terminal linker constructs. We showed that switching the *E. coli* expression strain from standard BL21(DE3) to NiCo21(DE3) and culture medium from Lysogeny to Terrific Broth were sufficient to boost the production of L_12_-*Am*NP to 25 mg/L (≥5-fold increase; for details, see the Supporting Information, Section S2.1). The effect of the α-helical linker position in the protein structure is probably complex, and more research beyond the scope of this study will be necessary for full clarification. However, at this stage, we were content with the result that both N- and C-terminal linker constructs could be expressed in useable yields with suitable adaptation of the expression conditions. The relevant constructs were produced efficiently and isolated in their expected sizes (*Lm*SP: Figure 2b; *Am*NP: Figures S4 and S5).

As shown in Figure 2c, the specific activity of *Lm*SP-L_6_ (248 ± 6 U/mg; *N* = 3) was only slightly lower compared to that of the native enzyme (i.e., C-terminally His-tagged *Lm*SP; 270 ± 19 U/mg; *N* = 3). By contrast, although it was possible to isolate L_7_-*Lm*SP (60.4 kDa; Figure 2b), its specific activity was only 19% that of the native enzyme (Figure 2c). Note: The N-terminally His-tagged *Lm*SP has the same specific activity as the reference *Lm*SP (Figure 2c) with a His-tag at the C-terminus. The N-terminus sits in a groove of the *Lm*SP structure while the C-terminus is exposed fully (Figure 1a). Appendage of the α-helical linker thus may have been more easily accommodated structurally and, during protein folding, at the C-terminus. The notion is corroborated by molecular modeling results (see later) that revealed negative, hence favorable, nonbonded interaction energies between the enzyme and the C-terminally placed linkers. The linker fusions *Lm*SP-L_14_ (63.8 kDa) and *Lm*SP-L_19_ (66.1 kDa) were thus prepared (Figure 2b) and they were obtained in useful expression yields (Figure 2c). Within the series of linker fusions, as shown in Figure 2c, the specific activity was not affected by the linker length. These three linker fusions of *Lm*SP thus provided a suitable set of enzymes for further immobilization studies.

Turning to *Am*NP, the specific activities (37 °C) of the linker constructs (L_12_-*Am*NP: ∼17 U/mg; *Am*NP-L_14_: ∼11 U/mg; *Am*NP-L_17_: ∼24 U/mg) were within a ∼3-fold range of the corresponding linker-free constructs (N- or C-terminally His-tagged enzyme; 38–39 U/mg; Figure 2c). However, the position of the linker affected the storage stability of the enzyme. *Am*NP-L_14_ and *Am*NP-L_17_ were fully stable over 2 weeks at −20 °C. L_12_-*Am*NP was inactivated (≥80%) within 1 week under identical conditions (Supporting Information, Section S2.1). We concluded at this point that C-terminal fusion of the linker(s) was the more promising strategy to proceed with both *Lm*SP and *Am*NP.

### Molecular Modeling of Linker Conformations

**Figure 3:**
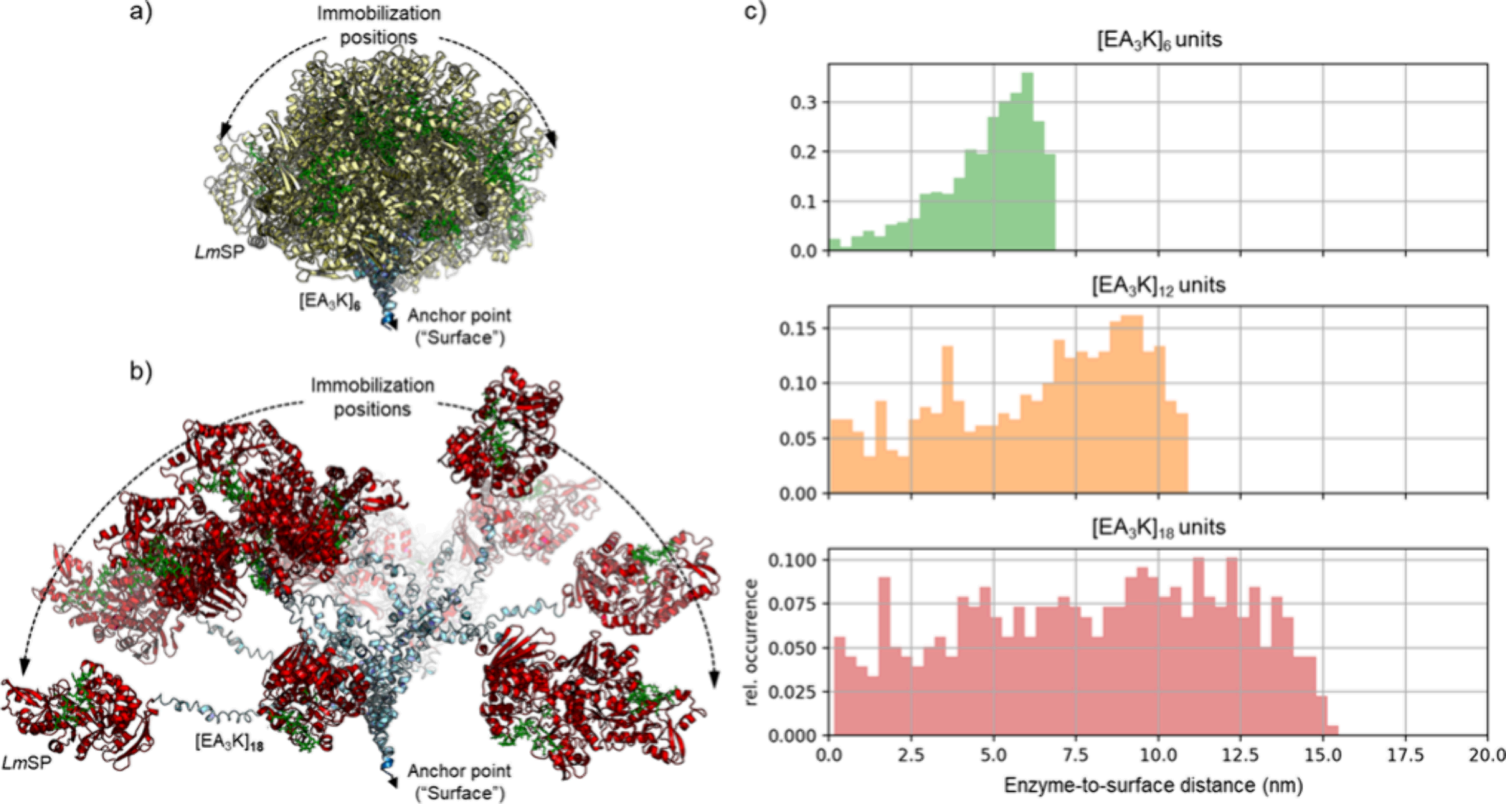
Molecular modeling results. Modeling of the enzyme-linker constructs positioning on the “surface” for (a) *Lm*SP-L_6_ and (b) *Lm*SP-L_18_, via anchor point (A_3_LE) for surface tethering; and (c) the distribution of enzyme-to-surface distance modeled for the tethered constructs containing linker in varied [EA_3_K] units.

Molecular dynamics simulations of the single EA_3_K fragment were performed and the resulting structures were classified into 55 clusters (for details, see the Supporting Information, Section S1.4). The three primary clusters accounted for approximately 60% of all of the simulated structures. These selected clusters encompassed contributions from the majority of replicate simulations (at least 7 out of 10 replicates), demonstrating their effective representation of the simulated trajectories. Their central member structures and overlay of the three central member structures are shown in Figure 1b. It is remarkable that this very small fragment quite persistently occurs in conformations of right-handed α-helices, suggesting a certain degree of stability. Differences between the three central member structures are in the local curvature of the helical axis, as the overlay of the three demonstrates. The depiction of the three central member structures in Figure 1b reveals a flip in the backbone’s ψ angle at the C-terminal end, which further contributes to the curvature observed in the global structure. Based on the central member structures of the single EA_3_K fragment (Figure 1b), the longer linkers were modeled in increments of *n* = 6 using a hierarchical clustering approach and applying energy minimization in each fusion step (see the Experimental section). The iterative process yielded structure models of three linkers (*n* = 6, 12, and 18), each including the terminal A_3_LE peptide that is joined to the His-tag in the experimental constructs. The modeled linker structures were fused to the C-terminus of *Lm*SP and the resulting models of the enzyme-linker fusion were aligned on the A_3_LE peptide, considering that this peptide serves as the common anchor point for surface tethering in the modeled population of immobilized enzyme conformers. Results are shown in Figure 3 (panel a: *Lm*SP-L_6_; panel b: *Lm*SP-L_18_) and Figure S6 (*Lm*SP-L_12_). The small differences in the local curvature of the EA_3_K peptides observed in Figure 1b, add up to considerable curvature in the *n* = 6, 12, and 18 constructs. The conformational space sampled by the enzyme-linker fusions relative to the invariant position on the surface gets broader as the linker length or *n* increases. The corresponding distributions of enzyme-to-surface distances are compared in Figure 3c. For *Lm*SP-L_6_, the distance distribution is centered between 5.5 and 6.5 nm (median: 5.2 nm; mode: 5.8, 6.1 nm) and exhibits left (negative) skewness. The maximum distance of 6.9 nm is consistent with a fully elongated [EA_3_K]_6_ linker of type #1 α-helix structure (Figure 1b). With increasing length of the linker, the enzyme-to-surface distances are no longer centered in the preferred range of values. This is seen best for the *Lm*SP-L_18_ conformers that populate distances between around 2 and 15 nm with almost identical frequencies (probabilities). The maximum distance are 10.9 and 15.5 nm for *Lm*SP-L_12_ and *Lm*SP-L_18_, respectively. This is shorter than expected from the double and triple distances of the elongated [EA_3_K]_6_ linker and indicates that the modeled conformers of the longer linkers exhibit increased degrees of curvature (Figure 3a,b).

### Enzyme Immobilization

Immobilization was performed on macroporous beads of hydrophilic agarose material that exhibit low nonspecific binding of the protein. The mean pore size is ∼30 nm,^71^ which is big enough to provide access into the pores for all enzyme constructs used. The Ni-NTA groups generally show a strong interaction with the His-tag. To assess the notion that the enzymes used are tethered to Ni-NTA agarose selectively via their His-tag, we analyzed immobilization of the native *Lm*SP on beads depleted of Ni^2+^ and further examined whether enzyme lacking the His-tag binds to the regular (metal-loaded) Ni-NTA agarose. Experiments performed under the stringent conditions of the highest amount of carrier used (10 mg/mL) showed that binding of the native *Lm*SP to the Ni^2+^-depleted NTA carrier was at the limit of detection (*Y* ≤ 1%), whereas it was almost complete (*Y* ≥ 93%) to the metal-loaded Ni-NTA carrier. Examined under the same conditions, *Lm*SP harboring N-terminal Strep-tag II did not bind to the Ni-NTA carrier (*Y* ≤ 3%). These results strongly support the contention that His-tag interaction with the surface Ni-NTA group drives the adsorption of *Lm*SP onto the solid carrier. Nonspecific binding of the *Lm*SP without the involvement of the His-tag is thus reasonably excluded. Of note, the evidence does not rule out the possibility of secondary surface interactions developed by the enzyme once it has become tethered via its His-tag.

**Figure 4:**
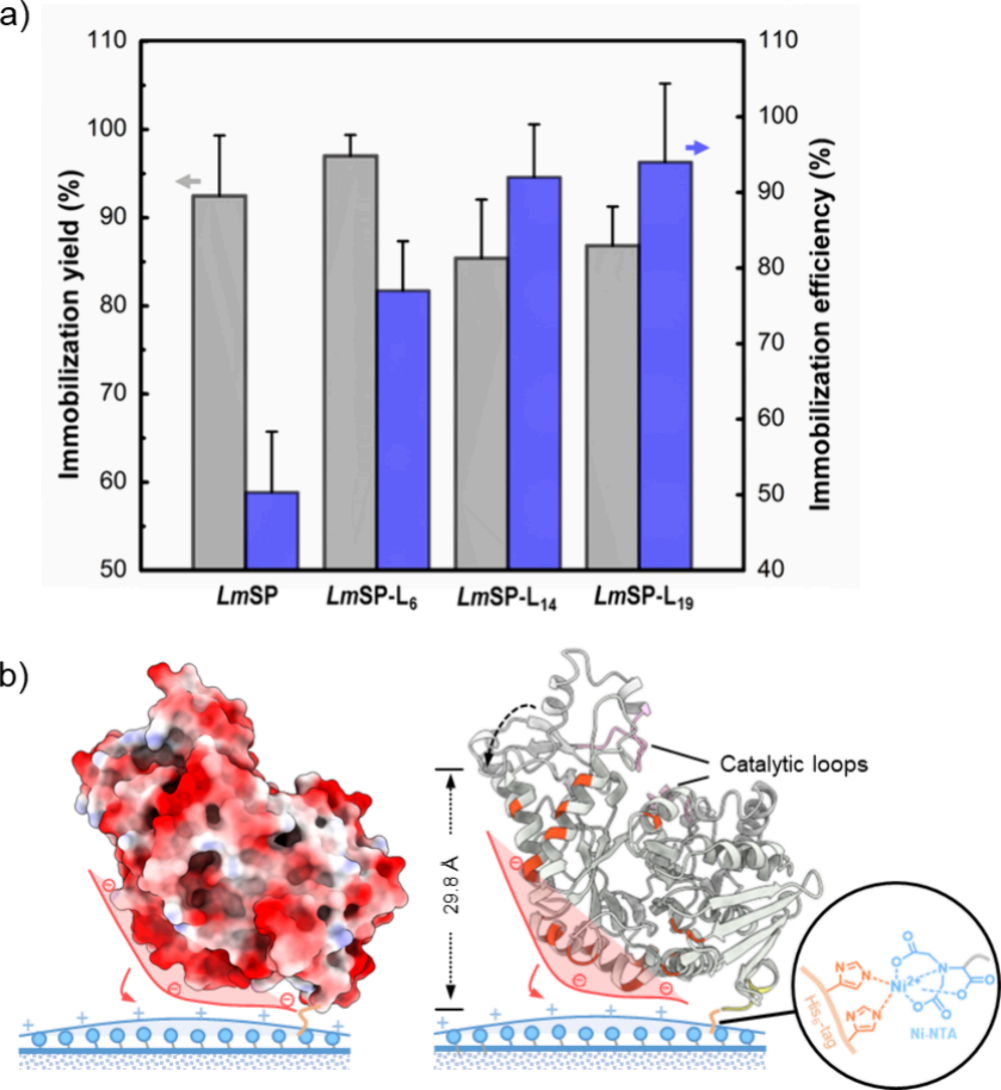
Immobilization of the *Lm*SP-linker constructs. (a) Immobilization yield *Y* (%; *N* ≥ 3) and efficiency η (%; *N* = 3) of native *Lm*SP and its linker fusions at the carrier loading of 10 mg/mL; (b) speculated electrostatic interactions between surface and native *Lm*SP when immobilized through the C-terminal His-tag. The enzyme is illustrated (by Chimera X) with an electrostatic potential surface and cartoon representation in the left and right panel, respectively. Negatively and positively charged residues are colored red and blue (Coulombic values: minimum −28.29, maximum 13.59), and charge-neutral residues are shown in white. The amino acids with negatively charged side chains (i.e., aspartic acid and glutamic acid) on the longitudinal region of enzyme are colored in orange red.

Experiments performed at variable carrier concentration (1–10 mg dry mass/mL) showed the remarkable result that the different linkers had only small effects on decreasing the binding affinity of the enzyme. At the highest carrier loading used (Figure 4a), the immobilization yield *Y* of the *Lm*SP-linker fusions converged at values between 85% and 97%, largely independent of the linker length. The *Y* of the native *Lm*SP was comparable at ∼93% (±7%; *N* = 5). At low carrier concentrations, the difference in *Y* between the native enzyme and the linker fusions was larger (up to 25%) and the trend appeared that longer linkers gave weaker binding (Figure S7). The immobilization efficiency η revealed an opposite trend: the native enzyme involved a relatively low value of η (50% ± 8%; *N* = 3) whereas the presence of a linker gave substantial improvement (Figure 4a). The value of η increased gradually with a greater length of linker. Immobilized preparations of *Lm*SP-L_14_ and *Lm*SP-L_19_ involved a near-perfect η value of 100% that is reached when the specific activity of the enzyme tethered to the surface is the same as in the solution.

A value of η below unity can reflect a continuum of states of immobilized enzymes between the extremes of a heterogeneous population of fully active and completely inactive enzymes, with η reporting the active portion of the enzyme, and a uniform population of configurationally distorted enzymes that exhibit lowered activity in the extent of η.^14^ The view of the heterogeneous enzyme population appears more intuitive and is also supported by the aggregate evidence from single-molecule studies of immobilized enzymes.^24−26^ Based on an ensemble-averaged parameter of immobilized enzyme activity such as η, however, a mechanistic distinction cannot be clearly achieved. We nonetheless examined structural reasons for the activity loss of *Lm*SP upon immobilization. Figure 4b visualizes the probable orientation of *Lm*SP for the His-tag-directed interaction with the solid surface. Besides enabling metal-affinity binding, the Ni-NTA groups also impart positive surface charge. The protein surface of *Lm*SP is mostly negatively charged. Assuming a limited degree of rotational freedom of the surface-tethered enzyme, as indicated in Figure 4b, the immobilized *Lm*SP will probably develop additional interactions with the surface based on complementary ionic charges. Not only can these interactions contribute to enhanced carrier binding affinity but they will likely also cause conformational distortions in the immobilized enzyme. The extent of activity loss as a consequence of such distortions requires speculation beyond the evidence here reported. However, Figure 4b identifies a plausible path of structural rearrangements that connects the protein region in contact with the solid surface to the enzyme's active site.

Immobilization studies of the *Am*NP offer additional insights. The *Am*NP with C-terminal His-tag was immobilized (0.5 mg of protein on 10 mg of carrier) in near-quantitative yield (95% ± 0.6%; *N* = 3) and almost complete retention of the efficiency of the soluble linker-free enzyme (η = ∼95%). The immobilization yield of *Am*NP-L_17_ was slightly lower (73% ± 2.5%; *N* = 3). However, the immobilized *Am*NP-L_17_ was as active (η = ∼100%; *N* = 3) as the soluble enzyme (for details, see the Supporting Information, Section S2.2). The idea of the α-helical linker as a spacer between the immobilized enzyme and the carrier surface is supported. As observed for *Lm*SP, an increased distance from the surface promotes the retention of the enzyme activity in the immobilized *Am*NP.

### Surface Effects on Catalysis Revealed by Temperature Kinetic Studies

Phosphorolysis of sucrose by the sucrose phosphorylases proceeds in two catalytic steps via a covalent glucosyl enzyme intermediate.^42,43^ The first catalytic step (enzyme glycosylation from sucrose) is rate-limiting for the *k*_cat_ of the enzyme from *Bifidobacterium longum*.^41^ High similarity among sucrose phosphorylases not only in their active-site structure but also in the actual value of the *k*_cat_ (= ∼10^2^ s^–1^) suggests a conserved localization of the rate-limiting step. Here, we determined the temperature dependencies of the *k*_cat_ of the *Lm*SP-linker fusions in the soluble and immobilized form. The corresponding *k*_cat_ temperature dependencies of the linker-free *Lm*SP were also determined and used as a reference. The temperature range (10–35 °C) was selected carefully to exclude the effects of enzyme inactivation at elevated temperatures.^44^ Plotted in semilog form, all temperature profiles were linear (Figure S8), supporting a fit with eq 1. The obtained thermodynamic activation parameters are summarized in Table 1. They provide an energetic description of the reactions catalyzed by each enzyme preparation. Note that the observable *k*_cat_ of the immobilized enzymes includes the effect of the immobilization efficiency η.

**Table 1:** Thermodynamic Activation Parameters for Phosphorolysis of Sucrose by Soluble and Immobilized Enzymes at 30 °C

**enzymes**	*k*_**cat**_**(s**^**–1**^**)**	**Δ***H*^**‡**^(kJ/mol)	**Δ***S*^**‡**^(J/(mol·K))	**Δ***G*^**‡**^(kJ/mol)^a^
soluble				
*Lm*SP	251 ± 17	54 ± 3.3	–24 ± 11	61
*Lm*SP-L_6_	248 ± 20	53 ± 4.1	–27 ± 14	61
*Lm*SP-L_14_	261 ± 31	52 ± 4.0	–26 ± 14	60
*Lm*SP-L_19_	265 ± 19	56 ± 2.8	–15 ± 10	60
immobilized				
*Lm*SP	87 ± 12	80 ± 3.7	54 ± 12	63
*Lm*SP-L_6_	207 ± 20	65 ± 3.7	12 ± 13	61
*Lm*SP-L_14_	220 ± 18	62 ± 3.7	5.0 ± 13	61
*Lm*SP-L_19_	284 ± 21	52 ± 2.1	–26 ± 7.0	60

a Calculated with the relationship Δ*G*^‡^ = Δ*H*^‡^ – *T*Δ*S*^‡^, using *T* = 303.15 K (30 °C).

Besides high similarity (±6%) in their *k*_cat_ values, the soluble enzymes were effectively identical in the activation parameters associated with their reactions. The Δ*H*^‡^ estimate was the same for all enzymes within the limit of error. As expected, the Δ*S*^‡^ was estimated at lower confidence than the Δ*H*^‡^. The Δ*S*^‡^ was essentially identical for all enzymes (mean: −19 ± 6.0 J/(mol·K)). The results strongly support the conclusion made earlier that the linkers do not affect the catalysis of *Lm*SP in solution, irrespective of their length.

Immobilization induced a clear linker dependency of the activation parameters for the reaction catalyzed. The *Lm*SP without linker involved the most pronounced change when comparing reactions by soluble and immobilized enzymes. For the reaction at 30 °C, the uptake of heat was increased strongly in the immobilized enzyme (ΔΔ*H*^‡^ = 26 kJ/mol), and the entropic contribution (−*T*Δ*S*^‡^) to the overall Gibbs free energy of activation (Δ*G*^‡^) changed from being positive for the soluble enzyme (7.3 kJ/mol) to negative (−16.4 kJ/mol) for the immobilized enzyme. The Δ*G*^‡^ associated with the reaction was, however, unchanged (∼61 kJ/mol). The immobilized *Lm*SP-linker fusions involved a distinct change in enthalpy–entropy partitioning within the frame of the largely invariant Δ*G*^‡^. Within the series of variable linker lengths that the three enzymes represent, the Δ*H*^‡^ and Δ*S*^‡^ associated with the *k*_cat_ of the immobilized enzyme gradually changed in the direction of the soluble enzyme, only to become identical in the longest linker fusion *Lm*SP-L_19_. These results strongly support the contention that increased distancing from the solid surface confers in solution-like native properties of enzyme catalysis to the immobilized *Lm*SP.

Results of the temperature-dependent studies of soluble and immobilized preparations of *Am*NP and *Am*NP-L_17_ are summarized in Table 2. In the linker-free *Am*NP, the Δ*H*^‡^ at 30 °C increased by 8 kJ/mol (ΔΔ*H*^‡^ was positive) for the reaction of the immobilized enzyme compared to the soluble enzyme. The Δ*G*^‡^ value was unchanged. Using *Am*NP-L_17_, the Δ*H*^‡^ for the reaction of the immobilized enzyme at 30 °C was lowered by ∼4 kJ/mol compared to the reaction of the soluble enzyme (Table 2). The negative value of ΔΔ*H*^‡^ was not well-defined statistically (Table 2), yet the important point in comparison to the linker-free *Am*NP is that ΔΔ*H*^‡^ upon immobilization was clearly not positive. This result aligned with the pattern observed for the linker fusions of *Lm*SP, where increased linker length restored enzyme behavior to a more native-like state. Although Δ*G*^‡^ was invariant for all reactions, Δ*H*^‡^ for the soluble enzyme reactions differed considerably between *Am*NP and *Am*NP-L_17_ (ΔΔ*H*^‡^ = 14 kJ/mol). Compensation by the activation entropy (-*T*ΔΔ*S*^‡^ = −13 kJ/mol) might be interpreted in terms of higher reactant-state conformational flexibility of the linker-free *Am*NP as compared to *Am*NP-L_17_ (as discussed below).

**Table 2:** Thermodynamic Activation Parameters for Synthesis of Nigerose by Soluble and Immobilized Enzymes at 30 °C

**enzymes**	*k*_**cat**_**(s**^**–1**^**)**	**Δ***H*^**‡**^(kJ/mol)	**Δ***S*^**‡**^(J/(mol·K))	**Δ***G*^**‡**^(kJ/mol)^a^
soluble				
*Am*NP	41 ± 2.4	36 ± 4.6	–96 ± 7.9	65
*Am*NP**-**L_17_	32 ± 2.1	50 ± 4.2	–53 ± 15	66
immobilized				
*Am*NP	43 ± 1.3	44 ± 1.5	–69 ± 5.2	65
*Am*NP-L_17_	30 ± 1.3	46 ± 2.8	–65 ± 9.6	66

a Calculated with the relationship Δ*G*^‡^ = Δ*H*^‡^ – *T*Δ*S*^‡^, using *T* = 303.15 K (30 °C).

**Figure 5:**
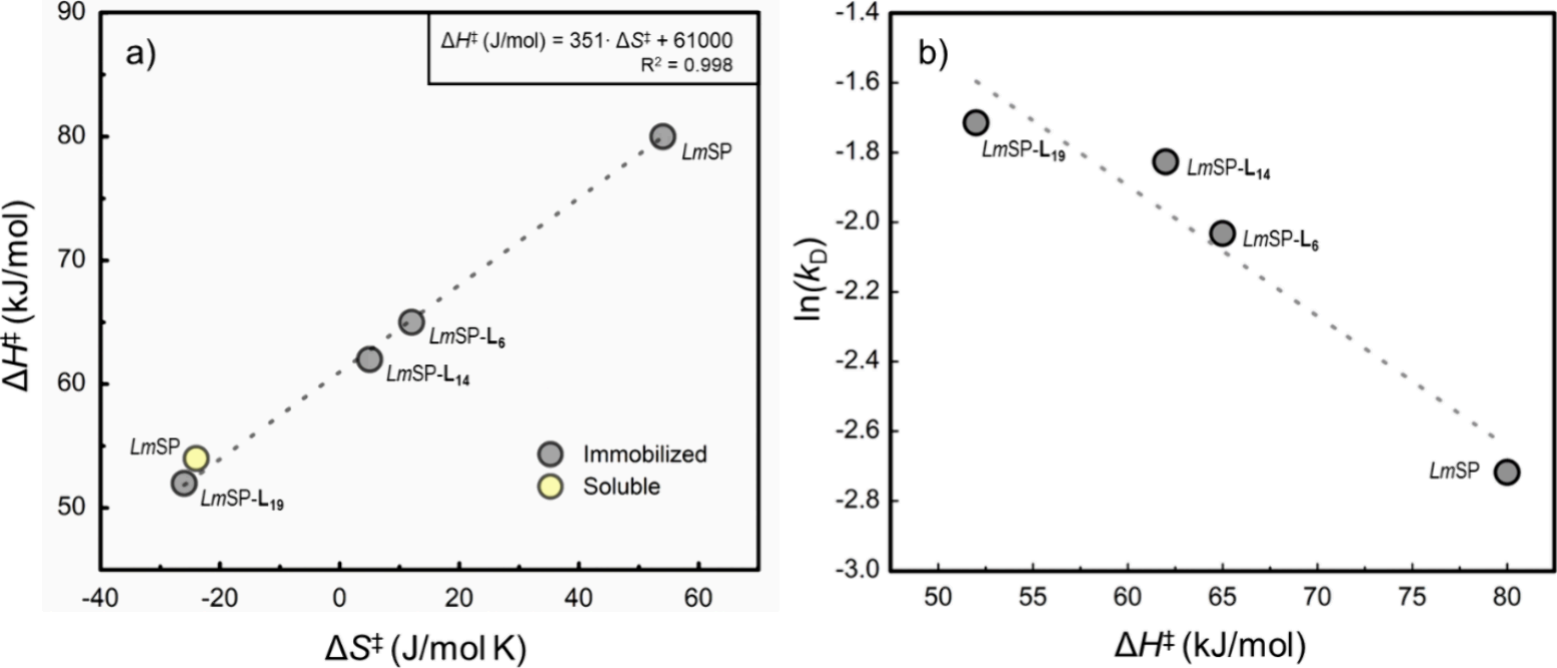
Enthalpy–entropy partitioning and kinetic stability in immobilized preparations of *Lm*SP constructs. (a) Plot of enthalpy (Δ*H*^‡^) against entropy (Δ*S*^‡^) and (b) semilogarithmic (ln) plot of deactivation constant (*k*_D_) against Δ*H*^‡^ from the immobilized *Lm*SP and the three linker-fusions. The averaged data of Δ*H*^‡^, Δ*S*^‡^ and *k*_D_ are illustrated without SD for clarity. The linear fits obtained are represented as dashed lines.

Focusing on the results for *Lm*SP, the constancy of Δ*G*^‡^ while Δ*H*^‡^ and Δ*S*^‡^ are changing suggests enthalpy–entropy compensation.^72^ Plotting Δ*H*^‡^ against Δ*S*^‡^ of the immobilized constructs revealed a notably linear correlation (*R*^2^ coefficient = 0.998), with the regression line intercepting at around 61 kJ/mol, representing the Δ*G*^‡^ (Figure 5a). The physical origin of enzymatic enthalpy–entropy compensation has been the subject of considerable debate,^73−76^ and the contribution from differential entropic activation is particularly challenging to explain in terms of the molecular interactions involved.^58^ MD/empirical valence bond (EVB) simulations of enzymatic temperature-rate profiles, and comparison of these profiles for orthologous enzymes with different optimum temperatures,^77−79^ provide important insights pertinent to the interpretation of the results obtained here (Figure 5a). It is known from experiments that cold-active psychrophilic enzymes (e.g., citrate synthase,^77^ trypsin,^78,79^ alcohol dehydrogenase^80^) involve an activation-free energy partitioning in their reactions different from that of their warm-active mesophilic orthologs. In particular, the cold-active enzymes appear to shift part of their activation-free energy from enthalpy to entropy. The MD/EVB simulations reproduce the experimental effect on enthalpy–entropy partitioning and clarify its origin from the structural flexibility of the protein surface.^58^ While the active-site core is similarly rigid in cold- and warm-adapted enzymes (e.g., trypsin), certain surface regions of the psychrophilic ortholog show enhanced enrichment in backbone mobility, resulting in a greater softness of the effective surface potential which includes the bound waters.^77−79^ The evidence that enzyme surface rigidity modulates the activation enthalpy–entropy balance immediately suggests an explanation for the observed change in activation-free energy partitioning in immobilized preparations of *Lm*SP, depending on the enzyme-surface distance controlled by spacer linker length. Enzymes positioned closer to the solid surface will arguably experience a higher degree of molecular organization (“rigidification”) at their surface. With a harder protein surface potential, the reactant configurational space will decrease, which may lead to enthalpy–entropy compensation by a more positive value of *T*Δ*S*^‡^. An alternative view is that the immobilized rigid *Lm*SP must relax for efficient catalysis through the additional uptake of heat under compensation by a gain in entropy. Plausible reasons for enzyme surface rigidification are direct interactions with the solid carrier as well as associations with the interfacial layer of water molecules. The water layer immediately adjacent to the solid surface exhibits enhanced water organization,^28,29^ resulting in increased microviscosity that in turn may restrict backbone motions in surface regions of the enzyme. The proposed scenario holds that the rigidifying interfacial interactions (surface positional restraints) are lost gradually as the *Lm*SP is placed at an increased distance from the solid surface, which leads to the original enthalpy–entropy partitioning for the enzymatic reaction in solution to become re-established. The successive distancing of the enzyme from the solid surface may involve analogous effects on protein surface restraint (in an opposite direction though) as were discovered by Åqvist and co-workers in MD/EVB simulations of the cold-adapted trypsin.^78,79^ The authors showed that upon successive restraining the atomic motion from the protein surface inward, the trypsin gradually acquired the characteristics of activation-free energy partitioning of the mesophilic enzyme.^78,79^

It is important to clearly distinguish molecular-level effects of enzyme-surface interaction on enzyme activity, as analyzed here, from well-known diffusional effects on activity treated in numerous publications of immobilized enzymes.^12,81^ Compared to the nanometer distance variation used in our experiments, diffusional effects arise at a much larger length scale (typically ≥10 μm, using agitated particles of the size employed here)^12^ and they affect the enzyme activity only indirectly. They are compound of equilibrium effects on liquid–solid phase partitioning of the substrates and kinetic effects on substrate mass transfer through the Nernst diffusion layer surrounding the solid particle.^81^ Besides considerations of length scale as mentioned above, diffusional effects are also excluded rigorously due to substrate concentrations used that were fully saturated for *Lm*SP at the steady state. The η value of ∼1 for the immobilized preparations of *Lm*SP-L_14_ and *Lm*SP-L_19_ proves the point. Similar conclusions can be extended to *Am*NP.

A harder surface potential of immobilized enzymes is likely to be intimately connected to stability.^58^ [Note: the effect discussed here must be distinguished from relationships between activity and stability (e.g., trade-off between the two^61^) discussed in other works on enzyme immobilization.^9−11,13−15^] We confirm this notion with the evidence that the kinetic stability of immobilized *Lm*SP (measured as apparent first-order inactivation rate constant *k*_D_ at 40 °C; plot profiles in Figure S9) decreased with increasing length of linker, as shown in Figure 5b. The overall change in *k*_D_ was not large (2.6-fold; 0.07 to 0.18 h^–1^; Table S6) but involved a clear dependence on the linker length in the way expected. It was interesting that immobilization rendered the *Lm*SP less stable (∼40-fold increase in *k*_D_) than it was in solution (*k*_D_ = 0.002 h^–1^). Intuitively, protein tethering is expected to result in stabilization or no effect on stability, and enzyme immobilization is generally performed with the idea of stabilizing the activity. However, several studies have shown that tethering can indeed lead to protein destabilization, presumably because the unfolded state ensemble of protein is stabilized in the tethered protein.^82,83^ Considering that *Lm*SP has been immobilized by other methods with a substantial gain in kinetic stability compared to the soluble enzyme,^45^ the current evidence of enzyme destabilization by His tag-directed selective immobilization on agarose can represent a relevant counterpart, potentially useful to explore mechanistic factors of immobilized enzyme stability in future research.

Lastly, we discuss the observation that the effect of surface tethering on the thermodynamic activation parameters was more pronounced in *Lm*SP than in *Am*NP. The difference in protein mass (*Lm*SP: 56.8 kDa; *Am*NP: 85.2 kDa) may provide a tentative explanation. Figure S10 shows a possible orientation of the His-tag-tethered *Am*NP on the carrier surface (for details, see the Supporting Information, Section S2.2). The estimated distance from the enzyme active site to the surface is larger for *Am*NP (∼40 Å) than for *Lm*SP (∼30 Å; Figure 4). The proposed molecular rigidification due to contact with the solid surface may thus be more relevant for *Lm*SP.

## Conclusions

Using α-helical rigid linkers of tunable spacer length, a fusion protein approach for the site-controlled, directed immobilization of *Lm*SP at an adjustable distance from the solid carrier surface was developed. The experimental method was critical to the study of elusive surface effects on immobilized enzyme catalysis. Interactions of *Lm*SP with the solid surface, including the interfacial layer of water molecules attached to the surface, result in enthalpy–entropy compensation with part of the overall activation-free energy shifted from entropy to enthalpy. The effect is explained by restrained surface mobility in the immobilized compared to the soluble enzyme: a hardened protein surface is thought to give a higher activation enthalpy for the chemical reaction, which is compensated by a smaller loss of entropy as the reaction barrier is climbed. Increasing the enzyme distance to the solid surface gradually resets the enthalpy–entropy balance to that of the enzymatic reaction in solution. Protein rigidification has often been postulated to explain enzyme stabilization on immobilization, yet its possible role in the energetics of immobilized enzyme catalysis was entirely unknown. The fundamental insight obtained here suggests rigid linker-based control of protein–surface distance as an engineering strategy to optimize the activity characteristics of surface-bound enzymes. Fusion of *Lm*SP to linkers of suitable length (L_14_, L_19_) results in the near-complete retention of specific activity and thermodynamic activation parameters of the original enzyme in solution. This level of activity control is hard to achieve with reported methods of enzyme immobilization.^13,14^ The studies performed with *Am*NP demonstrate the broader applicability of the fusion protein approach developed with *Lm*SP to other enzymes. It remains to be seen in which ways rigid linker-based tethering of enzymes at controlled distances to solid surfaces might become practical for enzyme immobilization in applied biocatalysis and bioanalysis to complement existing methodologies.^9−14^ Avoiding enzyme-surface interactions that negatively impact the activity characteristics of the immobilized enzyme in an uncontrollable or negative manner can be important in many enzyme applications. A method to position enzymes, or proteins, in general, at controlled distances from a solid surface is important in biophysics to explore the interfacial behavior of these biomolecules.

## Supporting Information Available

Sucrose phosphorylase catalyzed reactions for fine chemicals synthesis (Scheme S1); primers used for the preparation of linker constructs from *Lm*SP and *Am*NP (Table S1 and S2); enzyme concentration measured by Bradford and UV method (Table S3); [EA_3_K]_*n*_ linkers used for the construction of fusion enzymes (Table S4) and tagged enzymes (Table S5); identification of central member structure of EA_3_K unit in MD simulation (Figure S1); structure overview of proline-rich linkers (Figure S2); structure of *Am*NP (Figure S3); preparation of linker constructs of *Am*NP (Figure S4 and S5); molecular modeling of linker construct tethered on “surface” (Figure S6); carrier amount on enzyme immobilization (Figure S7); temperature kinetic profiles (Figure S8); thermostability profiles and deactivation constant of enzymes (Figure S9 and Table S6); speculated positioning of immobilized *Am*NP (Figure S10) (PDF)
