# Supplementary material for: Keeping the Distance: Activity Control in Solid-Supported Sucrose Phosphorylase by a Rigid α-Helical Linker of Tunable Spacer Length
Source: ACS Catal. 2024 Nov 6;14(22):17090–102. doi: 10.1021/acscatal.4c05616 (PMC11574764; doi:10.1021/acscatal.4c05616)
Supplement: Supplementary file 1 — cs4c05616_si_001.pdf [file cs4c05616_si_001.pdf]

Supporting Information

Keeping the distance: activity control in solid-supported sucrose phosphorylase by a rigid α-helical linker of tunable spacer length

Chao Zhong[1], Anisha Vyas[1,2], Jakob D. H. Liu[3], Chris Oostenbrink[3], and Bernd Nidetzky[1,2,*]

[1] Institute of Biotechnology and Biochemical Engineering, Graz University of Technology, NAWI Graz, Petersgasse 12, 8010 Graz, Austria

[2] Austrian Centre of Industrial Biotechnology (ACIB), Krenngasse 37, 8010 Graz, Austria

[3] Institute of Molecular Modeling and Simulation, University of Natural Resources and Life Sciences (BOKU), Muthgasse 18, 1190 Vienna, Austria

* Corresponding author (B.N.); e-mail: bernd.nidetzky@tugraz.at

Table of Contents

1. Materials and Methods 3

S1.1 Genetic information of enzyme and peptide linkers 3

S1.2 Genetic construction of linker fusion constructs 6

S1.3 Enzyme expression and purification 6

S1.4 Molecular dynamics simulation of single EA_3K unit 7

2. Supplemental Results 7

S2.1 Preparation of *Am*NP and linker constructs 7

S2.2 Immobilization of *Am*NP and linker constructs 7

3. Supplemental Scheme and Figures 8

Scheme S1. Sucrose phosphorylase catalyzed reactions for the synthesis of fine chemicals. 8

Figure S1. Identification of central member structure of EA_3K unit 8

Figure S2. Structure overview of Proline-rich linker. 9

Figure S3. Modeled protein structure of *Am*NP. 9

Figure S4. Expression comparison of the linker constructs derived from *Am*NP. 10

Figure S5. Expression profile of L_{12}-*Am*NP construct. 10

Figure S6. Molecular modeling of tethered linker construct 11

Figure S7. Effect of carrier amount on enzyme immobilization 11

Figure S8. Temperature kinetic profiles of enzymes 12

Figure S9. Thermostability profiles of enzyme 13

Figure S10. Immobilization of *Am*NP on Ni-NTA functionalized surface. 14

4. Supplemental Tables 15

Table S1. Primers used for the construction of *Lm*SP-linker constructs 15

Table S2. Primers used for the construction of *Am*NP-linker constructs 15

Table S3. Comparison of enzyme concentration (mg/mL) measured by Bradford assay and UV absorbance at 280 nm 15

Table S4. Overview of $[EA_3K]_n$ peptide linkers for the construction of fusion enzymes 16

Table S5. Overview of $[EA_3K]_n$ peptide linkers for the conjugation of functional tags and enzymes.. 16

Table S6. Deactivation rate constant k_D of enzymes at 40 °C 16

5. References 17

1. Materials and Methods

S1.1 Genetic information of enzyme and peptide linkers

a) Sucrose phosphorylase from *Leuconostoc mesenteroides* (***Lm*SP**, GenBank No. BAA14344.1)

Amino acid sequence:

MEIQNKAMLITYADSLGKNLKDVHQVLKEDIGDAIGGVHLLPFFPSTGDRGFAPADYTRVDAAFGDWA
DVEALGEEYYLMFDFMINHISRESVMYQDFKKNHDDSKYKDFFIRWEKFWAKAGENRPTQADVDLIY
KRKDKAPTQEITFDDGTTENLWNTFGEEQIDIDVNSAIAKEFIKTTLEDMVKHGANLIRLDAFAYAVKKV
DTNDFFVEPEIWDTLNEVREILTPLKAEILPEIHEHYSIPKKINDHGYFTYDFALPMTTLYTLYSGKTNQLA
KWLKMSPMKQFTTLDTHDGIGVVDARDILTDDEIDYASEQLYKVGANVKKTYSSASYNNLDIYQINSTY
YSALGNDDAAYLLSRVFQVFAPGIPQIYYVGLLAGENDIALLESTKEGRNINRHYYTREEVKSEVKRPVV
ANLLKLLSWRNESPAFDLAGSITVDTPTDTTIVVTRQDENGQNKAVLTADAANKTFEIVENGQTVMSSD
NLTQN*

Nucleotide sequence:

ATGGAAATCCAGAATAAGGCCATGCTGATTACCTATGCAGATAGCCTGGGTAAAAATCTGAAAGATG
TTCATCAGGTGCTGAAAGAAGATATTGGCGACGCAATTGGTGGTGTTCATCTGCTGCCGTTTTTTCCG
AGCACCGGTGATCGTGGTTTTGCACCGGCAGATTATACCCGTGTTGATGCAGCATTTGGTGATTGGGC
AGATGTTGAAGCACTGGGTGAAGAATATTATCTGATGTTCGACTTCATGATCAACCACATTAGCCGTG
AAAGCGTTATGTATCAGGACTTCAAAAAAAACCATGATGATAGCAAATACAAAGACTTTTTCATCCG
CTGGGAAAAGTTTTGGGCAAAAGCCGGTGAAAATCGTCCGACACAGGCCGATGTTGATCTGATCTAT
AAACGCAAAGATAAAGCACCGACGCAAGAAATCACCTTTGATGATGGCACCACCGAAAATCTGTGG
AATACCTTTGGTGAAGAACAAATTGATATCGATGTGAATAGCGCGATCGCCAAAGAATTTATCAAAAC
CACACTGGAAGATATGGTGAAACATGGTGCAAATCTGATTCGTCTGGATGCATTTGCCTATGCCGTTA
AAAAAGTGGATACCAACGATTTTTTTGTGGAACCGGAAATTTGGGATACCCTGAATGAAGTTCGTGA
AATTCTGACACCGCTGAAAGCAGAAATTCTGCCGGAAATTCATGAACATTATAGCATCCCGAAAAAA
ATCAACGATCACGGCTATTTCACCTATGATTTTGCACTGCCGATGACCACACTGTATACCCTGTATAGC
GGTAAAACCAATCAGCTGGCAAAATGGCTGAAAATGAGCCCGATGAAACAGTTTACAACCCTGGAT
ACCCATGATGGTATTGGTGTTGTTGATGCCCGTGATATCCTGACCGATGATGAAATTGATTATGCAAGC
GAGCAGCTGTATAAAGTTGGTGCCAATGTGAAAAAAACCTATAGCAGCGCAAGCTATAACAACCTGG
ATATCTATCAGATTAACAGCACCTATTATAGCGCACTGGGTAATGATGATGCAGCCTATCTGCTGAGCC
GTGTTTTTCAGGTTTTTGCTCCGGGTATCCCGCAGATCTATTATGTTGGTCTGCTGGCAGGCGAAAAT
GATATTGCCCTGCTGGAAAGCACCAAAGAAGGTCGTAACATTAATCGCCATTATTACACCCGTGAAG
AAGTGAAAAGCGAAGTTAAACGTCCGGTTGTTGCCAATCTGCTGAAACTGCTGAGCTGGCGTAATG
AAAGTCCGGCATTTGATCTGGCAGGTAGCATTACCGTTGATACCCCGACCGATACCACCATTGTTGTT
ACCCGTCAGGATGAAAATGGTCAGAATAAAGCAGTTCTGACCGCAGATGCAGCAAACAAAACCTTT
GAAATTGTTGAAAACGGCCAGACCGTTATGAGCAGCGATAATCTGACCCAGAAT(**TAA**)

The vector **pQE-30** (Qiagen) and **pET21b(+)** containing *lmsp* was used for the expression of **N-** and **C-**terminally His-tagged *Lm*SP, respectively.[1-2] The **pASK-IBA7+** construct introduced Strep-tag II at the **N-**terminus of *Lm*SP.[3]

b) α-Helix-forming peptide linkers

Sequence of the peptide $[EA_3K]_n$ linkers and fusion to the C- or N-terminus of *Lm*SP are demonstrated as follows.

C-terminally fused linker

$[EA_3K]_6$

Amino acid sequence (residues from C-terminus of *Lm*SP are colored in blue):

(QN-)LAEAAAKEAAAKEAAAKEAAAKEAAAKEAAAKAAALE*HHHHHH**
Nucleotide sequence:
(CAGAAT)CTGGCGGAGGCGGCGGCGAAGGAAGCGGCGGCGAAAGAAGCGGCGGCGAAAGAGGCG
GCGGCGAAAGAGGCGGCGGCGAAAGAGGCGGCGGCGAAGGCGGCGGCGCTCGAGCACCACCACC
ACCACCAC**TGA**

$[EA_3K]_{14}$
Amino acid sequence (residues from C-terminus of *Lm*SP are colored in blue):
(QN-)LAEAAAKEAAAKEAAAKEAAAKEAAAKEAAAKEAAAKEAAAKEAAAKEAAAKEAAAKEAAA
KEAAAKEAAAKAAALE*HHHHHH**
Nucleotide sequence:
(CAGAAT)CTGGCGGAGGCGGCGGCGAAGGAAGCGGCGGCGAAAGAAGCGGCGGCGAAGGAAGCG
GCGGCGAAAGAAGCGGCGGCGAAAGAGGCGGCGGCGAAAGAGGCGGCGGCGAAGGAAGCGGCG
GCGAAAGAGGCGGCGGCGAAGGAAGCGGCGGCGAAAGAAGCGGCGGCGAAAGAGGCGGCGGCG
AAAGAGGCGGCGGCGAAAGAGGCGGCGGCGAAGGCGGCGGCGCTCGAGCACCACCACCACCACC
AC**TGA**

$[EA_3K]_{19}$
Amino acid sequence (residues from C-terminus of *Lm*SP are colored in blue):
(QN-)LAEAAAKEAAAKEAAAKEAAAKEAAAKEAAAKEAAAKEAAAKEAAAKEAAAKEAAAKEAAA
KEAAAKEAAAKEAAAKEAAAKEAAAKEAAAKEAAAKAAALE*HHHHHH**
Nucleotide sequence:
(CAGAAT)CTGGCGGAGGCGGCGGCGAAGGAAGCGGCGGCGAAAGAAGCGGCGGCGAAGGAAGCG
GCGGCGAAAGAAGCGGCGGCGAAAGAAGCGGCGGCGAAAGAGGCGGCGGCGAAGGAAGCGGCG
GCGAAAGAGGCGGCGGCGAAGGAAGCGGCGGCGAAAGAAGCGGCGGCGAAAGAGGCGGCGGCG
AAAGAGGCGGCGGCGAAAGAGGCGGCGGCGAAGGAAGCGGCGGCGAAAGAAGCGGCGGCGAAA
GAGGCGGCGGCGAAAGAGGCGGCGGCGAAAGAGGCGGCGGCGAAGGCGGCGGCGCTCGAGCACC
ACCACCACCACCAC**TGA**

N-terminally fused linker
$[EA_3K]_7$
Amino acid sequence (residues from N-terminus of *Lm*SP are colored in green):
M*HHHHHH*LAEAAAKEAAAKEAAAKEAAAKEAAAKEAAAKEAAAKAAA(-ME)
Nucleotide sequence:
ATGCACCACCACCACCACCACCTGGCGGAGGCGGCGGCGAAAGAGGCGGCGGCGAAGGAAGCGG
CGGCGAAAGAGGCGGCGGCGAAGGAAGCGGCGGCGAAAGAAGCGGCGGCGAAAGAAGCGGCGG
CGAAGGCGGCGGCG(ATGGAA)

c) Nigerose phosphorylase from *Anaerosporobacter mobilis* (***Am*NP**; GenBank No. SHM33122.1)
Amino acid sequence:
MIADLKNWTIKESGFSEDKVTSNGNKFLCGNGYLGIRGTLEEFDKEYLPSINLAGIYDQVGNGWREPLN
APNGLYTRIKIDGVYYDLPKNEPVNHEQEVNYRHGIVTRITRWETRRGNITVTCERFAHYDKVHLICMR
YSILADFHADVEILTGIDGDVWDIHGPHYDQLLFEEEDIFTATGITHENKDRVAVAEEISVNHPYERKRKK
EGRKLLHRICLITEANKKIDLDKMVAIYTSKDCKEPEEAAKKEVREALQRGYEVCKSTHMNIWEEHWK
TAEIYIEGDPEAMEALNYSLYHLQCIAPRHSDSLSIAARGLSGQTYKGAVFWDTEMFMLDYFLYTQPEVA
KTLLRYRIDTLEGAKKKAESYGYEGAFYAWESQEGGYDACSDYNVTDVFTKRPMRTHFKDKQIHISAAI

VYGIMRYVKITGDQRFLEQGGIETILECAKFYDFLLVKKVHSDQYELHDVIGPDEYHERVNNNGYTNRM
AKFTFETAAKLLDDLMKFSKETIEKIEDNYDVERCKMDYREAAERIFIPKPDENGVLEQFDGYGKLEDA
SVEEVKGRLLHEKEYWGGAYGVASQTKVIKQADVVTWLTMFSEDFSEEVMLKNWRYYEPRTEHGSSL
SACMYALLACRCGMPQKAYSFFMKSASADLLPGGKEWAGLVYIGGTHPAAAGGAYMTAIQGFGGVYV
EDGELKVKPQLPEQWKKLRFTIKYQNQLYEIIETKDSAVINPL

Nucleotide sequence:

ATGATCGCCGATCTGAAAAACTGGACCATTAAAGAAAGCGGCTTTAGCGAAGATAAAGTGACCAGC
AATGGTAACAAATTTCTGTGCGGTAATGGTTATCTGGGTATTCGTGGCACCCTGGAAGAATTTGATAA
AGAATATCTGCCGAGCATTAACCTGGCAGGTATTTATGATCAGGTTGGTAATGGCTGGCGTGAACCGC
TGAATGCACCGAATGGTCTGTATACCCGTATCAAAATTGATGGCGTGTATTATGATCTGCCGAAAAAT
GAACCGGTGAACCATGAACAAGAGGTGAATTATCGTCATGGTATTGTTACCCGTATTACCCGTTGGGA
AACCCGTCGTGGCAATATTACCGTTACCTGTGAACGTTTTGCCCACTATGACAAAGTTCATCTGATTT
GCATGCGCTATAGCATTCTGGCAGATTTTCATGCCGATGTTGAAATTCTGACCGGTATTGATGGTGATG
TGTGGGATATTCATGGTCCGCACTATGATCAGCTGCTGTTTGAAGAAGAAGATATTTTCACCGCAACC
GGCATTACCCATGAAAATAAAGATCGTGTTGCAGTGGCAGAAGAAATCAGCGTTAATCATCCGTATG
AACGCAAACGTAAGAAAGAAGGTCGTAAACTGCTGCATCGTATTTGTCTGATTACCGAAGCCAACA
AGAAAATCGATCTGGATAAAATGGTGGCCATCTATACCAGCAAAGATTGTAAAGAACCGGAAGAAGC
AGCCAAGAAAGAGGTTCGCGAAGCACTGCAGCGTGGTTATGAAGTTTGTAAAAGCACCCACATGAA
CATCTGGGAAGAACATTGGAAAACCGCAGAAATCTATATTGAAGGTGATCCGGAAGCAATGGAAGC
ACTGAATTATAGCCTGTATCATCTGCAGTGTATTGCACCGCGTCATAGCGATAGCCTGAGCATTGCAG
CACGTGGTCTGAGTGGTCAGACCTATAAAGGTGCAGTGTTTTGGGATACCGAAATGTTCATGCTGGA
TTACTTTCTGTATACACAGCCGGAAGTTGCAAAAACCCTGCTGCGTTATCGTATTGATACACTGGAAG
GTGCCAAAAAGAAAGCAGAAAGCTATGGCTATGAAGGTGCATTTTATGCATGGGAAAGCCAAGAGG
GTGGCTATGATGCATGTAGCGATTATAATGTTACCGACGTGTTTACCAAACGTCCGATGCGTACCCATT
TTAAAGATAAGCAGATTCATATTAGCGCAGCCATTGTGTATGGTATTATGCGCTATGTGAAAATTACCG
GTGATCAGCGTTTTCTGGAACAAGGTGGTATTGAAACCATTCTGGAATGCGCCAAATTCTATGATTTT
CTGCTGGTGAAAAAGGTGCACAGCGATCAGTATGAACTGCATGATGTTATTGGTCCGGATGAATATC
ATGAACGCGTGAATAATAACGGCTATACCAATCGTATGGCCAAGTTTACCTTTGAAACCGCAGCAAA
ACTGCTGGATGATCTGATGAAATTTAGCAAAGAAACCATCGAGAAGATCGAGGATAATTATGATGTG
GAACGCTGCAAAATGGATTATCGTGAAGCAGCAGAACGTATCTTTATTCCGAAACCGGATGAAAATG
GCGTGCTGGAACAGTTTGATGGTTATGGTAAACTGGAAGATGCAAGCGTTGAAGAAGTTAAAGGTC
GTCTGCTGCACGAAAAAGAATATTGGGGTGGTGCCTATGGTGTTGCAAGCCAGACCAAAGTTATTAA
ACAGGCAGATGTTGTTACCTGGCTGACCATGTTTAGTGAAGATTTTTCTGAAGAGGTGATGCTGAAA
AATTGGCGCTATTATGAACCGCGTACCGAACATGGTAGCAGCCTGAGCGCATGTATGTATGCACTGCT
GGCATGTCGTTGTGGTATGCCGCAGAAAGCATATTCGTTCTTTATGAAAAGCGCAAGTGCCGATCTGC
TGCCTGGTGGTAAAGAATGGGCAGGTCTGGTTTATATTGGTGGTACACATCCGGCAGCAGCCGGTGG
TGCATATATGACCGCAATTCAAGGTTTTGGTGGTGTGTATGTTGAAGATGGTGAACTGAAAGTTAAAC
CTCAGCTGCCGGAACAGTGGAAAAAGCTGCGTTTTACCATCAAATATCAGAACCAGCTGTACGAGAT
CATCGAAACCAAAGATAGCGCAGTTATTAATCCGCTG(**TAA**)

The vector **pET21b(+)** containing the codon-optimized *amnp* gene was used to generate *Am*NP constructs with either N- or C-terminal His-tag. $[EA_3K]_n$ peptide linkers were introduced at the N- or C-terminus of *Am*NP. The linker sequences are not shown as they are similar to those used in the *Lm*SP constructs.

S1.2 Genetic construction of linker fusion constructs

The linker constructs of *Lm*SP and *Am*NP were prepared using Prolonged Overlap Extension-PCR (POE-PCR).[4] Based on the sequences from Section **S1.1**, a series of primers (see Table **S1**-**S2**) were designed and used for DNA multimer generation, which were then introduced into competent *Escherichia coli* BL21(DE3) cells. The LB-agar plates, supplemented with ampicillin (100 mg/L), were inoculated with the transformed *E. coli* cells and incubated overnight at 37°C. After incubation, individual colonies were selected and cultured in 10 mL of sterilized LB media containing ampicillin (100 mg/L) for overnight incubation at 37°C. Plasmid extraction was then performed using a Promega Wizard® DNA Extraction Kit according to the standard protocol. The isolated plasmids were sequenced prior to enzyme expression.

S1.3 Enzyme expression and purification

a) *Lm*SP & linker constructs

With the exception of the N-terminal Strep-tagged *Lm*SP, all *Lm*SP-derived enzymes were prepared as follows. *E. coli* BL21(DE3) cells harboring the respective plasmid were cultivated in 1-L baffled shaken flasks at 37°C and 110 rpm using LB media supplemented with 100 mg/L ampicillin. Enzyme expression was induced with 0.25 mM isopropyl β-D-1-thiogalactopyranoside (IPTG) at 18°C overnight. Cells were harvested (5,000 rpm, 4°C, 20 min), resuspended in buffer (50 mM NaH_2PO_4, 300 mM NaCl, pH 7.4) and disrupted by ultrasonication (Sonic Dismembrator Model 505, Fisher Scientific, Austria) with the protocol: 6 min with alternating 2 s pulse on/4 s pulse off at 60% amplitude. The enzymes were purified via N- or C-terminal His-tag using a HisTrap FF crude column (5 mL) on an ÄKTA Start system (Cytiva, Austria), with elution performed using a gradient concentration of imidazole (0.01 to 0.3 M) in phosphate buffer (50 mM NaH_2PO_4, 300 mM NaCl, pH 7.4).

For the N-terminal Strep-tagged *Lm*SP, *E. coli* Top10 cells harboring pASK-IBA7+ vectors were cultured in LB media (100 mg/L ampicillin), following the procedure described above. Enzyme expression was induced with 200 µg/L anhydrotetracycline at 25°C for 12 h. Cells were harvested (5,000 rpm, 4°C, 20 min), suspended in buffer (100 mM Tris, 150 mM NaCl, 10 mM EDTA, pH 8.0) and disrupted by ultrasonication: 5 min with alternating 5 s pulse on/10 s pulse off at 60% amplitude. The enzymes were purified via the N-terminal Strep-tag II using a StrepTrap XT column (5 mL) on an ÄKTA Start system, and eluted with 2.5 mM biotin.

The purified proteins were then desalted and concentrated using Vivaspin Turbo 10 kDa cut-off concentrator tubes (Sartorius Stedim, Austria) with storage buffer (50 mM NaH_2PO_4, 50 mM NaCl; pH 7.4).

b) *Am*NP & linker constructs

E. coli BL21(DE3) cells were used for the expression of N-/C-terminally His-tagged *Am*NP, as well as the N- or C-terminal linker constructs, following the procedure described above.

In addition, *E. coli* NiCo21(DE3) strains (New England Biolabs, MA, US) were used to express the N-terminal construct L_{12}-*Am*NP, to reduce contamination from *E. coli* proteins (GlmS, SlyD, ArnA, Can) in the immobilized metal affinity chromatography (IMAC) fractions. Cells carrying the plasmid encoding L_{12}-*Am*NP were cultured in 1-L baffled flasks at 37°C, 110 rpm, in Terrific Broth (TB) with 100 mg/L ampicillin. Expression was induced at an OD_{600} of ~1.0 with 0.25 mM IPTG, followed by overnight incubation at 18°C. Purification via the His-tag followed the protocol described above, with each eluted fraction analyzed by SDS-PAGE before desalting and concentrating. Note, the desalting step utilized a 50 mM MES/NaCl buffer (pH 7.2) as the storage buffer.

S1.4 Molecular dynamics simulation of single EA_3K unit

The simulation of the EA_3K fragments involved 10 independent runs, each starting from the same initial structure but using distinct random seeds. For each run, the trajectories were clustered individually. The resulting cluster analysis is visualized in a plot, which reveals the specific clusters visited by each simulation. In a combined cluster analysis, structures from all 10 simulations were grouped into 55 clusters. The three clusters (#**1-3**) were frequented by the majority (at least 7 out of 10) of the simulations, as shown in Figure **S1**.

2. Supplemental Results

S2.1 Preparation of *Am*NP and linker constructs

*Am*NP was selected as an additional model enzyme for linker construction. Accordingly, linker constructs $\mathbf{L_6}$**-/**$\mathbf{L_{12}}$**-***Am*NP (with N-terminal linkers of approximately 5 and 10 nm, respectively), and *Am*NP-$\mathbf{L_{14}}$**/**$\mathbf{L_{17}}$ (with C-terminal linkers of approximately 10 and 15 nm, respectively) were prepared.

The native *Am*NP (N- and C-terminally His-tagged), with a molecular weight of 85.2 kDa, yielded approximately 80 mg protein/L culture, as estimated from the purified fractions. C-terminal fusions (*Am*NP-L_{14}/-L_{17}) maintained similar expression levels, producing 60-80 mg/L culture. In contrast, N-terminal linker fusions (L_6- and L_{12}-*Am*NP) exhibited significantly reduced expression levels, with yields of < 5 mg/L culture. This disparity is illustrated in **Figure S4**: faint bands corresponding to the expected sizes of full-length N-terminal fusion proteins (88.3 and 91.1 kDa) were detected in whole-cell extracts, while distinct bands for full-length C-terminal fusion proteins (92.2 and 93.6 kDa) were visible in both whole-cell extracts and purified fractions.

Due to the low expression levels of N-terminal fusions, the host strain was switched to *E. coli* NiCo21(DE3) and expressed in TB medium. Consequently, L_{12}-*Am*NP was expressed with an improved yield of approximately 25 mg/L culture and was obtained relatively high purity (**Figure S5**).

S2.2 Immobilization of *Am*NP and linker constructs

By comparing immobilization yield across enzyme loadings from 0.25-1.5 mg, optimal conditions were identified using ~0.5 mg of enzyme per 10 mg (dry weight) carrier. Under these conditions, C-terminally His-tagged *Am*NP achieved an immobilization yield of 95% ± 0.6% ($N = 3$), while *Am*NP-L_{17} achieved a yield of 73% ± 2.5% ($N = 3$). Increasing the enzyme loading to 1.5 mg for the linker-fusion enzyme resulted in a decreased yield of 46%.

At enzyme loading of ~0.5 mg, the immobilized *Am*NP and *Am*NP-L_{17} exhibited catalytic efficiencies of 96% ± 0.9% and 97% ± 2.1% ($N = 3$), respectively. To elucidate the structural basis underlying the retained catalytic activity of linker-free *Am*NP upon immobilization, two possible explanations are proposed. First, the surface surrounding the C-terminus of *Am*NP is relatively neutral (**Figure S10**), which may reduce significant rotational restrictions or conformational changes of the enzyme when immobilized, minimizing additional interactions with the carrier that could negatively impact activity. Second, the catalytic loops (active site) of *Am*NP are positioned approximately 40 Å away from the surface upon immobilization. Given that the solid surfaces typically extend into liquid phase by ~10 Å, interference from the carrier surface is likely negligible. Consequently, the immobilized *Am*NP largely retains its catalytic efficiency.

Additionally, the presence of the linker may also contribute to the retention of catalytic properties in the tethered enzyme. Although the improvement in catalytic efficiency is modest as compared to native *Am*NP, the role of the linker is considered similar to that observed with *Lm*SP.

3. Supplemental Scheme and Figures

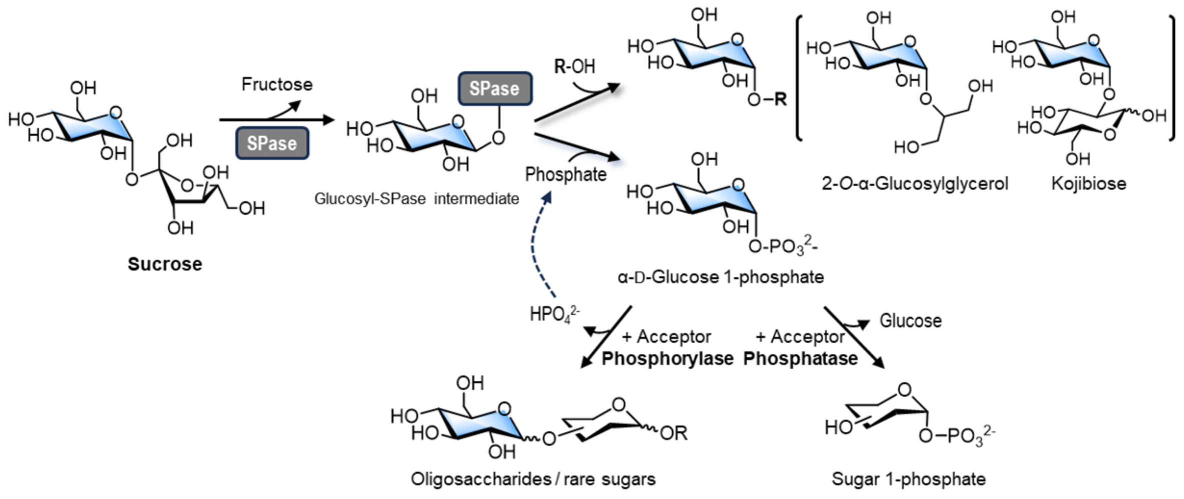

Scheme S1. Sucrose phosphorylase catalyzed reactions for the synthesis of fine chemicals. The glycosides and certain rare sugars can be synthesized via the transglucosylation reaction. The α-D-glucose 1-phosphate generated from phosphorolysis reaction can serve as a substrate for other enzymes to generate oligosaccharides and sugar 1-phosphate.

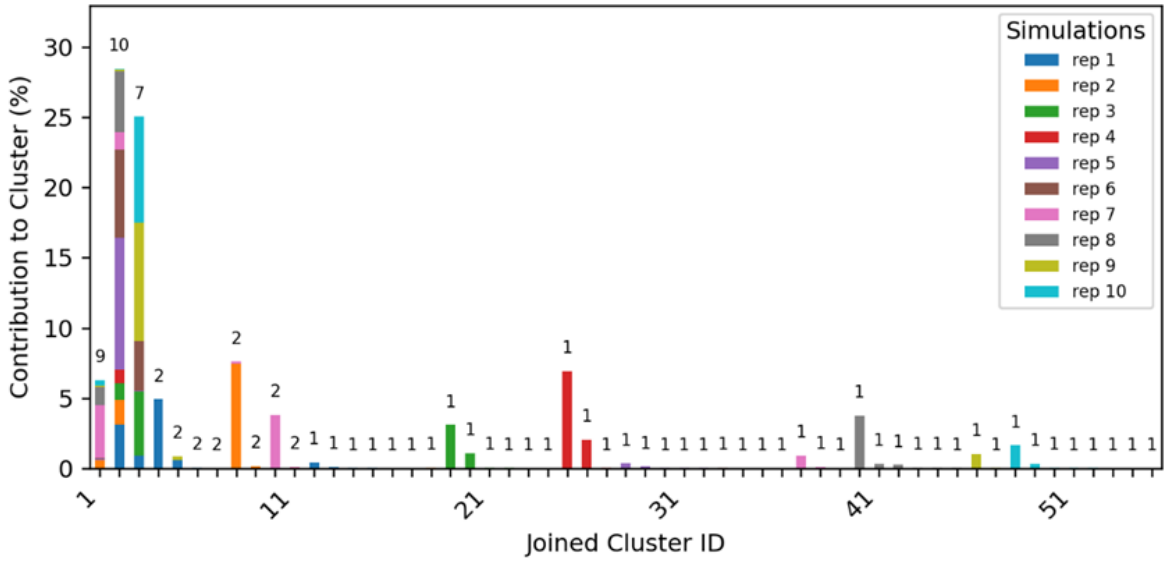

Figure S1. Identification of central member structure of EA3K unit. Distribution of the structure across joined clusters based on an RMSD matrix of the backbone atoms from all simulated trajectories in 10 replicate simulations. The numbers above the bars indicate the count of simulations contributing to each cluster.

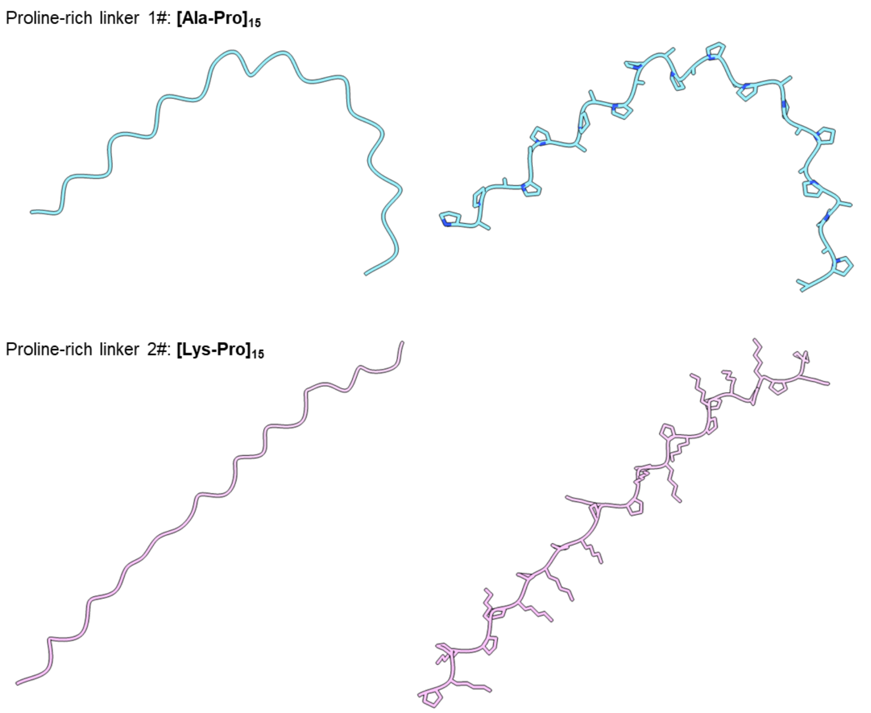

Figure S2. Structure overview of Proline-rich linker. The structural simulation of the two linkers, as visualized by Chimera X, was conducted using the SWISS-MODEL server (https://swissmodel.expasy.org/). These linkers are designed to impart structural rigidity, while controlling their conformation and maintaining precise distances presents challenges.

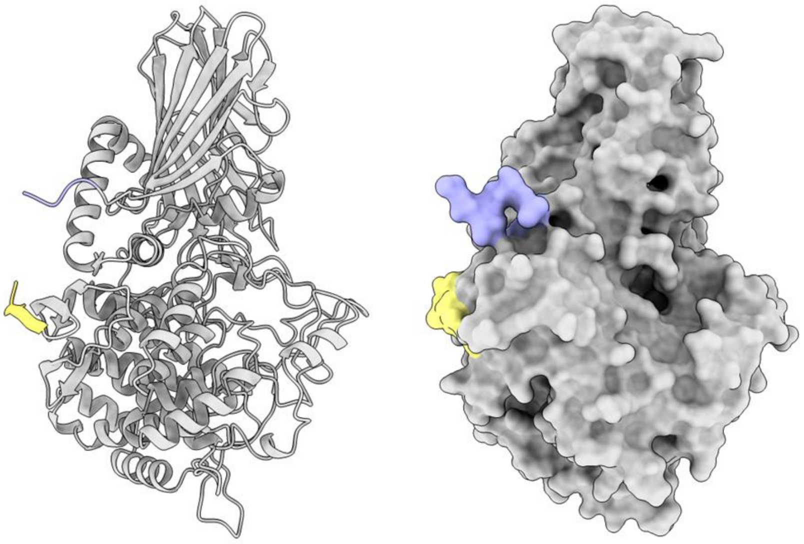

Figure S3. Modeled protein structure of *Am*NP. AlphaFold structure model (AF-A0A1M7HX72-F1) of the enzyme is illustrated (by Chimera X) as cartoon and surface structure in left and right panel, respectively. The N- and C-terminus of enzyme is colored in purple and yellow, respectively.

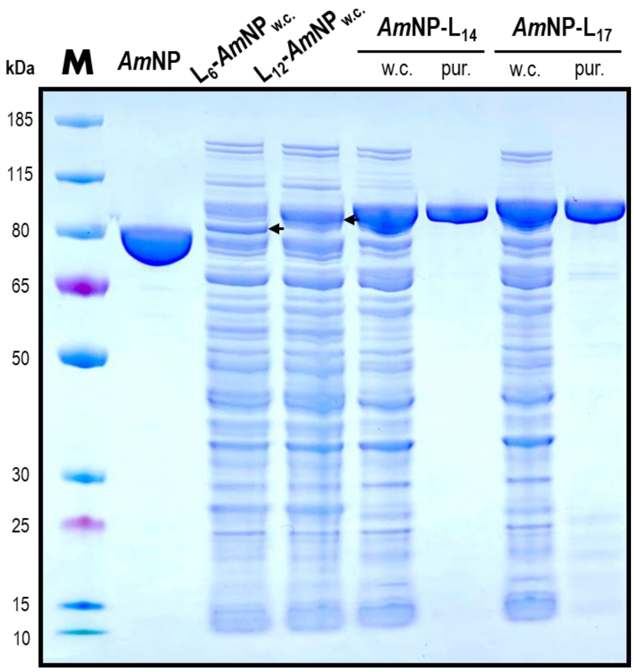

Figure S4. Expression comparison of the linker constructs derived from *Am*NP. Preparations of purified native *Am*NP and its N-/C-terminal linker fusion constructs. **M**, PageRuler^{TM} Plus Prestained Protein Ladder (10-185 kDa). The purified proteins migrated to positions in the gel consistent with their expected molecular masses. "w.c." indicates the whole-cell extract, and "pur." denotes the purified enzyme preparation. The arrow indicates potential bands corresponding to the N-terminal linker fusions of *Am*NP.

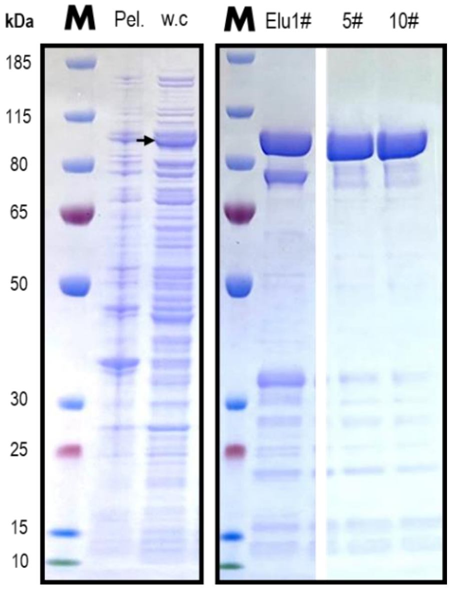

Figure S5. Expression profile of L12-*Am*NP construct. Preparations of L12-*Am*NP and its eluted fractions (1, 5, and 10#) during purification. **M**, PageRuler^{TM} Plus Prestained Protein Ladder (10-185 kDa). The purified protein migrated to a position in the gel consistent with its expected molecular mass (91.1 kDa). "Pel" refers to the pellet from cell lysate, "w.c." indicates the whole-cell extract, and "Elu. #" denotes the respective elution fraction number.

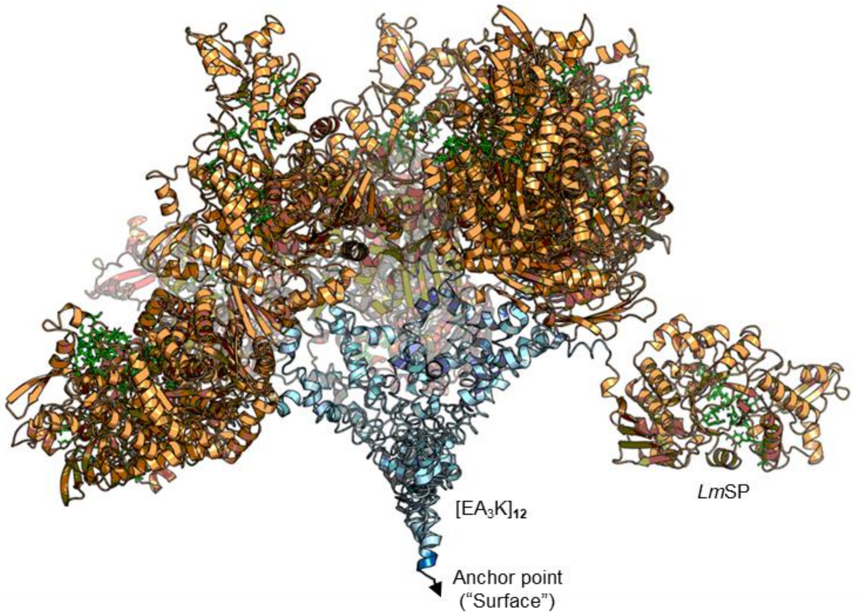

Figure S6. Molecular modeling of tethered linker construct. Modeling of *Lm*SP-L12 positioning on the "surface" via a single anchor point (A3LE) for surface tethering.

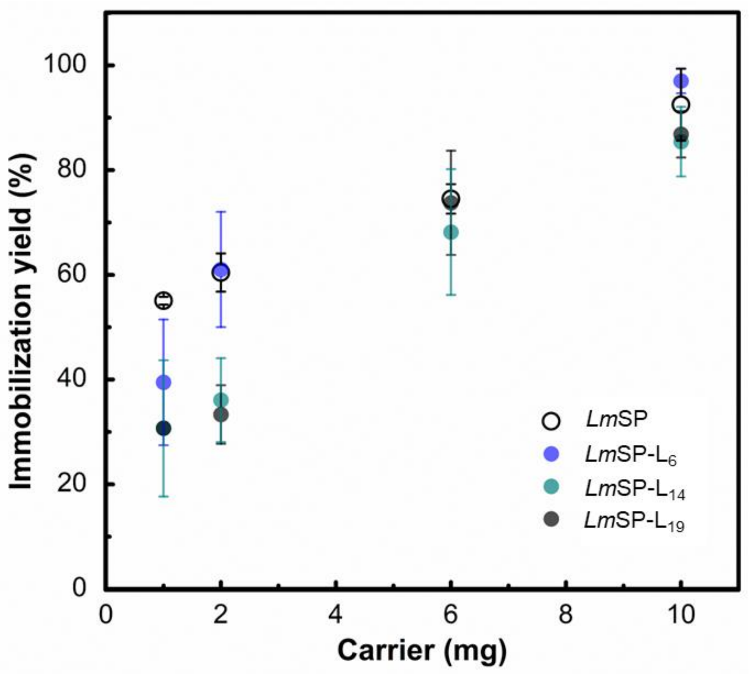

Figure S7. Effect of carrier amount on enzyme immobilization. Immobilization yield Y (%; N ≥ 3) of native *Lm*SP and its linker constructs (approximately 2 mg) onto varying amounts (dry mass; mg) of the carrier (Ni-NTA agarose beads). Averaged values are denoted as circles, accompanied by standard deviations represented as error bars.

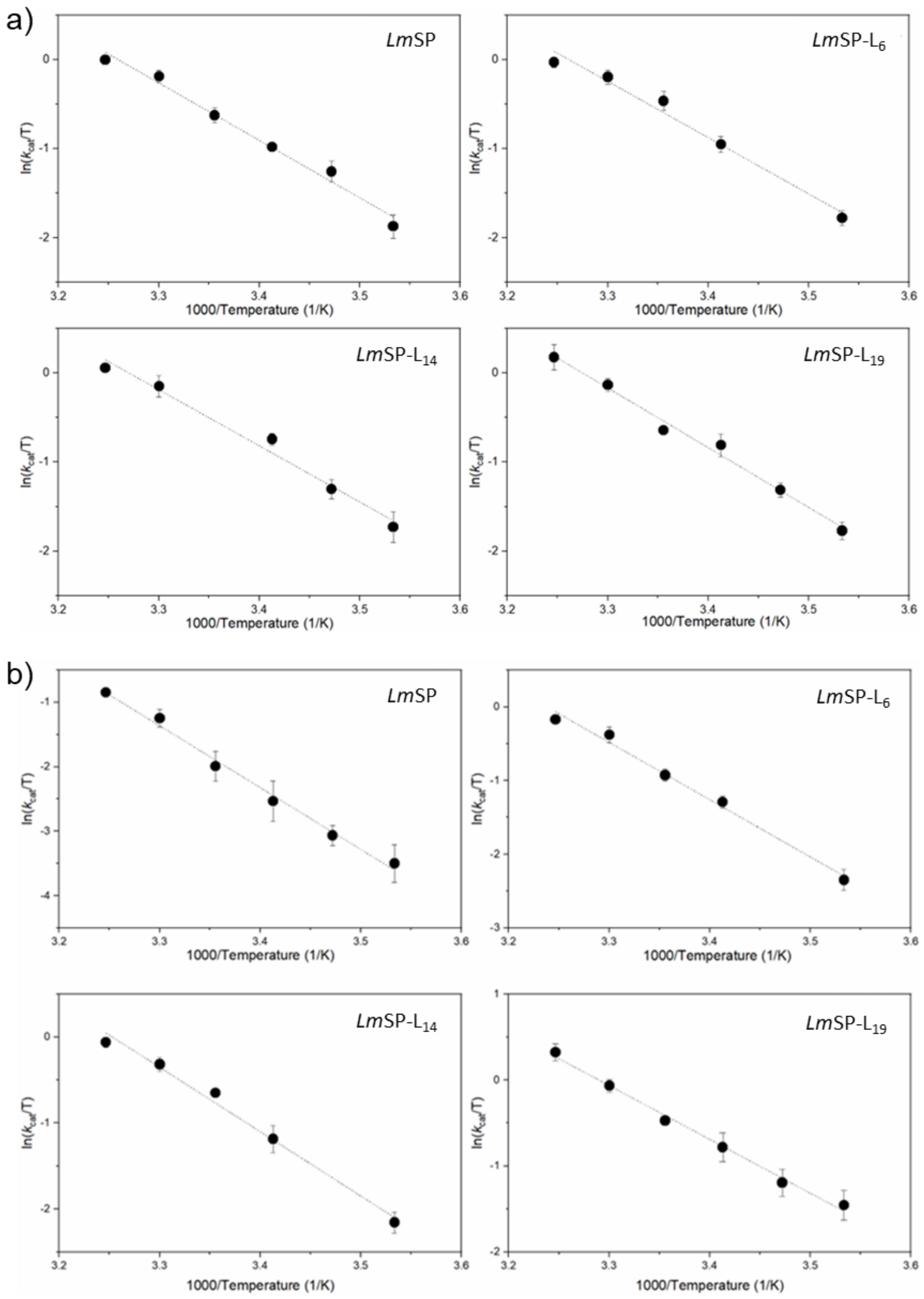

Figure S8. Temperature kinetic profiles of enzymes. Eyring plots showing the temperature dependence (i.e., 10-35°C) of *Lm*SP and its linker-constructs in **a)** soluble and **b)** immobilized form. Experimental data are presented in averages with associated standard deviations ($N = 3$). The linear fit is obtained with correlation of fit ≥ 0.98.

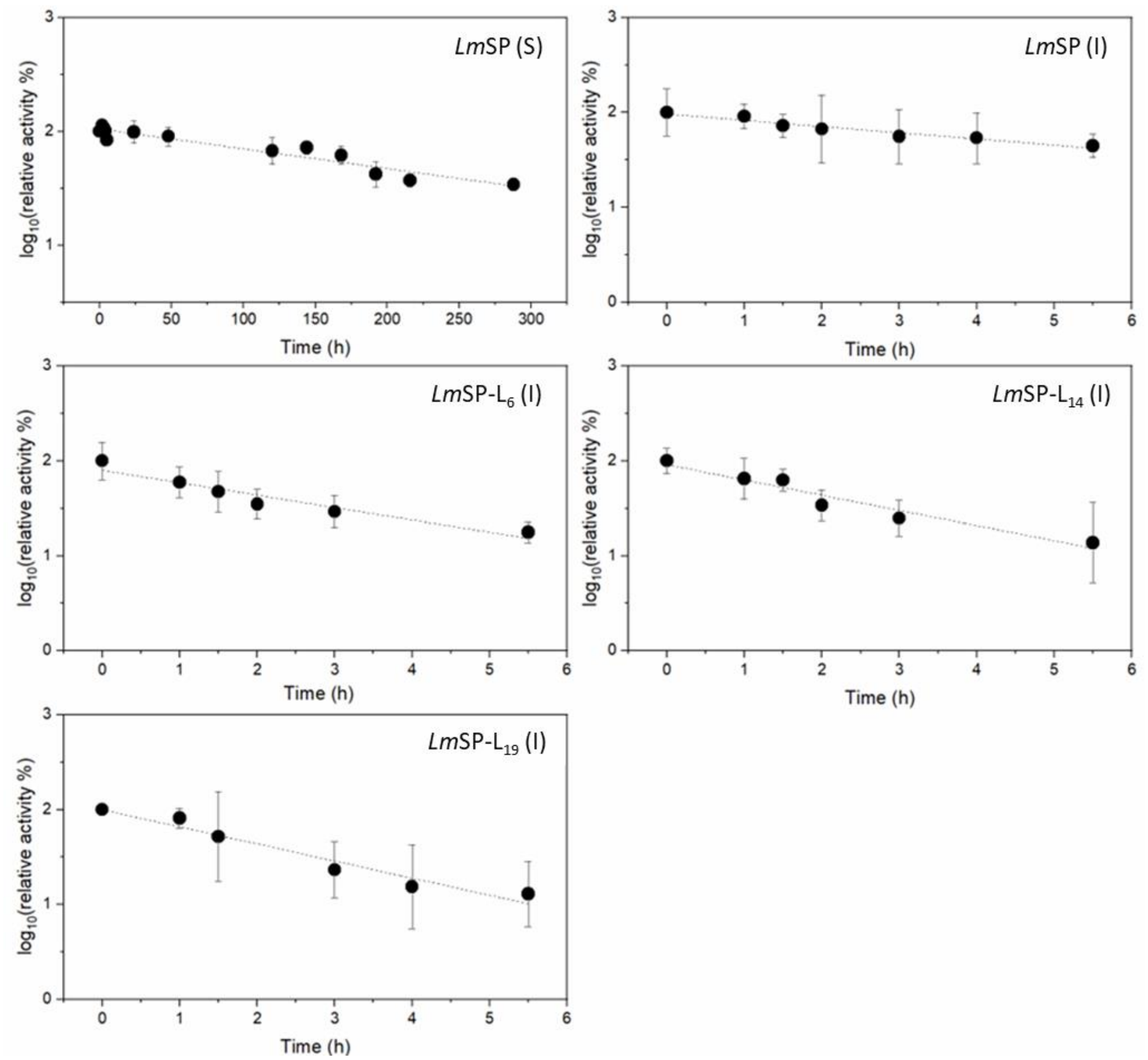

Figure S9. Thermostability profiles of enzyme. Linear semi-log plots of residual activity (%) versus incubation time (h) for the soluble (S) *Lm*SP and immobilized (I) enzymes at 40°C. The experimental data are presented in averages with associated standard deviations ($N = 3$). The linear fit is obtained with correlation of fit ≥ 0.92. The residual activity (%) is calculated as the ratio of the activity measured at a specific time point to the original activity. The stability test was conducted using an enzyme concentration of approximately 10 μg/mL.

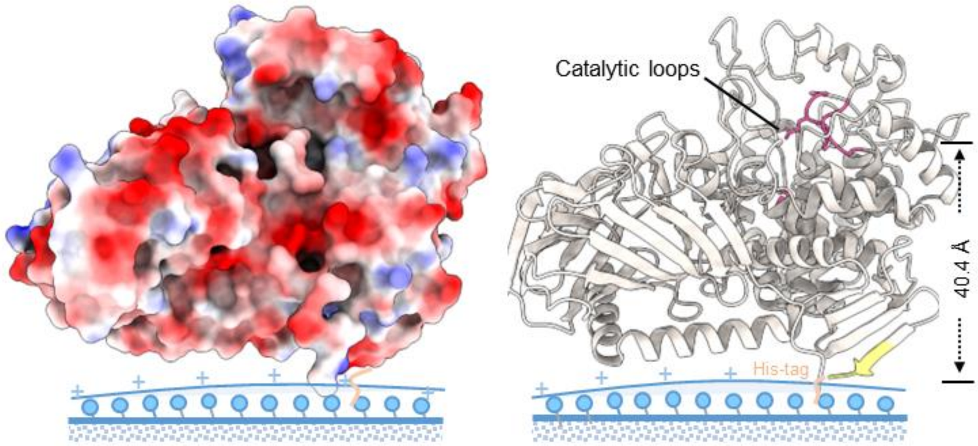

Figure S10. Immobilization of *Am*NP on Ni-NTA functionalized surface. Speculated orientation of *Am*NP on the carrier surface anchored via the C-terminal His-tag. The enzyme is depicted by Chimera X, with its electrostatic potential surface and cartoon representation shown in the left and right panels, respectively. The negatively charged residues are shown in red, and positively charged residues are in blue (Coulombic potential values: minimum -16.74, maximum 9.73), with charge-neutral residues represented in white. The vertical distance between the active site and surface, aligned with the level of the C-terminus (light-yellow colored) of *Am*NP, is estimated to be ~40 Å.

4. Supplemental Tables

Table S1. Primers used for the construction of *Lm*SP-linker constructs

Primers	Primer sequence	Plasmid of construct
*Lm*_C-terminal IF	ACTTTAAGAAGGAGATATACATATGGAAATCCAGAATAAGGCCATGCTG	pET21b_***Lm*SP-L$_6$/L$_{14}$/L$_{19}$**-Histag
*Lm*_C-terminal VR	CAGCATGGCCTTATTCTGGATTTCCATATGTATATCTCCTTCTTAAAGT	
*Lm*_C-terminal VF [a]	AGCAGCGATAATCTGACCCAGAATCTGGCGGAGGCGGCGGCGAAGGAAGC	
*Lm*_C-terminal IR	GCTTCCTTCGCCGCCGCCTCCGCCAGATTCTGGGTCAGATTATCGCTGCT	
*Lm*_N-terminal IF	GAAGCGGCGGCGAAGGCGGCGGCGATGGAAATCCAGAATAAGGCCATG	pET21b_Histag-**L$_7$-*Lm*SP**
*Lm*_N-terminal VR	CATGGCCTTATTCTGGATTTCCATCGCCGCCGCCTTCGCCGCCGCTTC	
*Lm*_N-terminal VF	AGCAGCGATAATCTGACCCAGAATTAACTCGAGCACCACCACCACCACC	
*Lm*_N-terminal IR	GGTGGTGGTGGTGGTGCTCGAGTTAATTCTGGGTCAGATTATCGCTGCT	

[a] Due to the repetitive nature of EA$_3$K sequence within the plasmid, the primer "*Lm*_C-terminal VF" may bind to multiple occurrences of the EA$_3$K sequence, potentially resulting in the amplification of shorter linker sequences than intended. In this instance, the [EA$_3$K]$_6$ sequence was amplified from the original sequence [EA$_3$K]$_7$ and used for the study.

Table S2. Primers used for the construction of *Am*NP-linker constructs

Primers	Primer sequence	Plasmid of construct
*Am*_C-terminal IF	CTTTAAGAAGGAGATATACATATGATGATCGCCGATCTGAAAAACTGG	pET21b_***Am*NP-L$_{14}$/L$_{17}$**-Histag
*Am*_C-terminal VR	CCAGTTTTTCAGATCGGCGATCATCATATGTATATCTCCTTCTTAAAG	
*Am*_C-terminal VF [a]	GATAGCGCAGTTATTAATCCGCTGCTGGCGGAGGCGGCGGCGAAGGAAG	
*Am*_C-terminal IR	CTTCCTTCGCCGCCGCCTCCGCCAGCAGCGGATTAATAACTGCGCTATC	
*Am*_N-terminal IF	GAGGCGGCGGCGAAGGCGGCGGCGATGATCGCCGATCTGAAAAACTGG	pET21b_Histag-**L$_6$/L$_{12}$-*Am*NP**
*Am*_N-terminal VR [a]	CCAGTTTTTCAGATCGGCGATCATCGCCGCCGCCTTCGCCGCCGCCTC	
*Am*_N-terminal VF	GATAGCGCAGTTATTAATCCGCTGTAACTCGAGCACCACCACCACCAC	
*Am*_N-terminal IR	GTGGTGGTGGTGGTGCTCGAGTTACAGCGGATTAATAACTGCGCTATC	

[a] Due to the repetitive nature of EA$_3$K sequence within the plasmid, the primer "*Am*_C-terminal VF" and "*Am*_N-terminal VR" may bind to multiple occurrences of the EA$_3$K sequence, resulting in the amplification of shorter linker sequences than intended. In this instance, the sequences [EA$_3$K]$_{6/12/17}$ were amplified from the [EA$_3$K]$_{7/14/19}$ sequence, respectively, and were used for the study.

Table S3. Comparison of enzyme concentration (mg/mL) measured by Bradford assay and UV absorbance at 280 nm

Enzymes	Bradford assay (*N*=2) [a]	UV absorbance assay (*N*=3) [b]
*Lm*SP	**20.4** ± 0.1	**21.1** ± 0.5
*Lm*SP-**L$_6$**	**34.1** ± 2.5	**36.3** ± 2.6
*Lm*SP-**L$_{14}$**	**14.4** ± 0.3	**15.9** ± 1.2
*Lm*SP-**L$_{19}$**	**35.9** ± 0.6	**36.4** ± 4.1
*Am*NP	**21.7** ± 0.9	**19.3** ± 0.5
**L$_{12}$**-*Am*NP	**3.3** ± 0.3	**2.6** ± 0.2
*Am*NP-**L$_{14}$**	**5.6** ± 0.7	**6.8** ± 0.1
*Am*NP-**L$_{17}$**	**5.5** ± 0.5	**5.5** ± 0.1

[a] Concentration was determined by RotiQuant reagent (Carl Roth) employing bovine serum albumin as standard.
[b] Concentration was determined from absorbance at 280 nm, utilizing the molar extinction coefficient (M^{-1} cm^{-1}) and molecular weight (Da) calculated by the ExPASy ProtParam tool (https://web.expasy.org/protparam/).

Table S4. Overview of $[EA_3K]_n$ peptide linkers for the construction of fusion enzymes

**Fusion enzymes** [a]	**Linker**	**Properties of fusion enzymes**	**Ref.**
Fluc-**L**-LRE	$-[EA_3K]_1-$	Exp: ☑; Act: reduced Fluc; 2-fold of LRE	5
Glucanase-**L**-xylanase	$-[EA_3K]_3-$	Exp: ☑; Act: 2-fold of Glu; retained Xyl	6
GDH-**L**-LeuDH	$-[EA_3K]_3-$	Exp: ☑; Act: retained for both	7
NOX-**L**-ADH	$-[EA_3K]_3-$	Exp: ☑; Act: 2-fold of NOX; 70% of ADH	8
Xylanase-**L**-mannanase & mannanase-**L**-xylanase	$-[EA_3K]_4-$	Exp: ☑; Act: retained mannanase; reduced xylanase	9
Arazyme-**L**-TGFαL_3	$-A[EA_3K]_4ALEA[EA_3K]_4A-$	Exp: ☑; Act: retained Arazyme	10
GCSF-**L**-GCSF (dimeric)	$-A[EA_3K]_4ALEA[EA_3K]_4A-$	Exp: insoluble (refolding needed); Act: half of monomer; 7-fold of $t_{1/2}$	11
Transferrin-**L**-hGH	$-A[EA_3K]_4ALEA[EA_3K]_4A-$	Exp: 2.4-fold; Act: 2-fold cell proliferation	12
Transferrin-**L**-GCSF	$-A[EA_3K]_4ALEA[EA_3K]_4A-$	Exp: 11.2-fold; Act: n.a.	12

[a] Enzyme modules for fusion: -**L**- denotes linker; Fluc, firefly luciferase; LRE, luciferin-regenerating enzyme; GDH, glucose dehydrogenase; LeuDH, leucine dehydrogenase; NOX, NADH oxidase; ADH, alcohol dehydrogenase; TGFαL_3, transforming growth factor alpha third loop; GCSF, granulocyte-colony stimulating factor; hGH, human growth hormone.
[b] Properties of the construct include: expression (exp.), activity (act.) and relevant ones.

Table S5. Overview of $[EA_3K]_n$ peptide linkers for the conjugation of functional tags and enzymes

**Enzyme construct** [a]	**Linker**	**Properties of the enzyme construct** [b]	**Ref.**
CBP-**L**-chitosanase	$-[EA_3K]_5-$	Exp: ☑; Act: ☑; Immo (chitin-column): ☑	13
MBP-**L**-GFP-**L**-heparinase I	$-[EA_3K]_3-$	Exp: ☑; Act: ☑; Immo (MBP-Trap column): ☑	14
SAP-**L**-GFP	$-[EA_3K]_5-$	Exp: ☑; Act: n.a.	15
SAP-**L**-polygalacturonate lyase	$-[EA_3K]_5-$	Exp: ☑; Act: ☑	16
scFv5-**L**-AviTag	$-[EA_3K]_3-$	Exp: ☑; Act: n.a.; Immo (streptavidin): ☑	17
VP1-**L**-SpyTag	$-[EA_3K]_3-$	Exp: ☑; Act: n.a.; 1.5-fold conjugation efficiencies of SpyTag with linker fused	18

[a] Functional tags are underlined: -**L**- denotes linker; CBP, chitin-binding protein; MBP, maltose binding protein, SAP, self-assembling amphiphilic peptides; AviTag (15 aa), prepared for biotinylated proteins; SpyTag (13 aa), for binding to SpyCatcher.
[b] Properties of the construct include: expression (exp.), activity (act.) and immobilization (carriers) if available.

Table S6. Deactivation rate constant k_D of enzymes at 40 °C

**Enzymes**	k_D **(h^{-1}; $N = 3$)** [a]
Immobilized	
*Lm*SP	**0.066** ± 0.007
*Lm*SP-$\mathbf{L_6}$	**0.131** ± 0.018
*Lm*SP-$\mathbf{L_{14}}$	**0.179** ± 0.016
*Lm*SP-$\mathbf{L_{19}}$	**0.180** ± 0.021
Soluble	
*Lm*SP	**0.002** ± 0.0002

[a] k_D (h^{-1}) is obtained from the slope (absolute value) of linear semi-log plots in **Figure S9**.

5. References

(1) Goedl, C.; Schwarz, A.; Minani, A.; Nidetzky, B. Recombinant sucrose phosphorylase from *Leuconostoc mesenteroides*: characterization, kinetic studies of transglucosylation, and application of immobilised enzyme for production of alpha-D-glucose 1-phosphate. *J. Biotech.* **2007,** *129* (1), 77-86, DOI: 10.1016/j.jbiotec.2006.11.019.

(2) Schwaiger, K. N.; Cserjan-Puschmann, M.; Striedner, G.; Nidetzky, B. Whole cell-based catalyst for enzymatic production of the osmolyte 2-*O*-α-glucosylglycerol. *Microb. Cell Fact.* **2021,** *20* (1), 79, DOI: 10.1186/s12934-021-01569-4.

(3) Wildberger, P.; Luley-Goedl, C.; Nidetzky, B. Aromatic interactions at the catalytic subsite of sucrose phosphorylase: Their roles in enzymatic glucosyl transfer probed with Phe52→Ala and Phe52→Asn mutants. *FEBS Lett.* **2011,** *585* (3), 499-504, DOI: 10.1016/j.febslet.2010.12.041.

(4) Zhong, C.; You, C.; Wei, P.; Zhang, Y. H. Simple cloning by prolonged overlap extension-PCR with application to the preparation of large-size random gene mutagenesis library in *Escherichia coli*. *Methods in molecular biology* **2017,** *1472*, 49-61, DOI: 10.1007/978-1-4939-6343-0_4.

(5) Sun, X.; Tang, X.; Hu, R.; Luo, M.; Hill, P.; Fang, B.; Xu, C. Biosynthetic bifunctional enzyme complex with high-efficiency luciferin-recycling to enhance the bioluminescence imaging. *Int. J. Biol. Macromol.* **2019,** *130*, 705-714, DOI: 10.1016/j.ijbiomac.2019.03.036.

(6) Lu, P.; Feng, M. G. Bifunctional enhancement of a beta-glucanase-xylanase fusion enzyme by optimization of peptide linkers. *Appl. Microbiol. Biotechnol.* **2008,** *79* (4), 579-87, DOI: 10.1007/s00253-008-1468-4.

(7) Liao, L.; Zhang, Y.; Wang, Y.; Fu, Y.; Zhang, A.; Qiu, R.; Yang, S.; Fang, B. Construction and characterization of a novel glucose dehydrogenase-leucine dehydrogenase fusion enzyme for the biosynthesis of L-tert-leucine. *Microb. Cell Fact.* **2021,** *20* (1), 3, DOI: 10.1186/s12934-020-01501-2.

(8) Wu, X.; Zhang, C.; Xing, X. H.; Yun, Z.; Zhao, L.; Wu, Q. Construction and characterization of novel bifunctional fusion proteins composed of alcohol dehydrogenase and NADH oxidase with efficient oxidized cofactor regeneration. *Biotechnol. Appl. Biochem.* **2022,** *69* (4), 1535-1544, DOI: 10.1002/bab.2225.

(9) Guo, N.; Zheng, J.; Wu, L.-S.; Tian, J.; Zhou, H.-B. Engineered bifunctional enzymes of endo-1,4-β-xylanase/endo-1,4-β-mannanase were constructed for synergistically hydrolyzing hemicellulose. *J. Mol. Catal. B Enzym.* **2013,** *97*, 311-318, DOI: 10.1016/j.molcatb.2013.06.019.

(10) Mehrab, R.; Sedighian, H.; Sotoodehnejadnematalahi, F.; Halabian, R.; Fooladi, A. A. I. A comparative study of the arazyme-based fusion proteins with various ligands for more effective targeting cancer therapy: an in-silico analysis. *Res. Pharm. Sci.* **2023,** *18* (2), 159-176, DOI: 10.4103/1735-5362.367795.

(11) Mickiene, G.; Dalgediene, I.; Dapkunas, Z.; Zvirblis, G.; Pesliakas, H.; Kaupinis, A.; Valius, M.; Mistiniene, E.; Pleckaityte, M. Construction, purification, and characterization of a homodimeric granulocyte colony-stimulating factor. *Mol. Biotechnol.* **2017,** *59* (9), 374-384, DOI: 10.1007/s12033-017-0026-7.

(12) Amet, N.; Lee, H. F.; Shen, W. C. Insertion of the designed helical linker led to increased expression of tf-based fusion proteins. *Pharm. Res.* **2009,** *26* (3), 523-8, DOI: 10.1007/s11095-008-9767-0.

(13) Wu, Y. J.; Fan, C. Y.; Li, Y. K. Protein purification involving a unique auto-cleavage feature of a repeated EAAAK peptide. *J Chromatogr B Analyt Technol Biomed Life Sci* **2009,** *877* (31), 4015-21, DOI: 10.1016/j.jchromb.2009.10.009.

(14) Huang, Z.; Ye, F.; Zhang, C.; Chen, S.; Chen, Y.; Wu, J.; Togo, M.; Xing, X.-H. Rational design of a tripartite fusion protein of heparinase I enables one-step affinity purification and real-time activity detection. *J. Biotech.* **2013,** *163* (1), 30-37, DOI: 10.1016/j.jbiotec.2012.09.016.

(15) Zhao, W.; Liu, S.; Du, G.; Zhou, J. An efficient expression tag library based on self-assembling

amphipathic peptides. *Microb. Cell Fact.* **2019,** *18* (1), 91, DOI: 10.1186/s12934-019-1142-9.
(16) Zhao, W.; Du, G.; Liu, S. An efficient thermostabilization strategy based on self-assembling amphipathic peptides for fusion tags. *Enzyme Microb. Technol.* **2019,** *121*, 68-77, DOI: 10.1016/j.enzmictec.2018.11.004.
(17) Ikonomova, S. P.; Le, M. T.; Kalla, N.; Karlsson, A. J. Effect of linkers on immobilization of scFvs with biotin-streptavidin interaction. *Biotechnol. Appl. Biochem.* **2018,** *65* (4), 580-585, DOI: 10.1002/bab.1645.
(18) Boonyakida, J.; Khoris, I. M.; Nasrin, F.; Park, E. Y. Improvement of modular protein display efficiency in Spytag-implemented norovirus-like particles. *Biomacromolecules* **2023,** *24* (1), 308-318, DOI: 10.1021/acs.biomac.2c01150.
